# Transcriptome analysis of inflammation-related gene expression in endothelial cells activated by complement MASP-1

**DOI:** 10.1038/s41598-017-09058-8

**Published:** 2017-09-05

**Authors:** Endre Schwaner, Zsuzsanna Németh, Péter K. Jani, Erika Kajdácsi, Márta L. Debreczeni, Zoltán Doleschall, József Dobó, Péter Gál, János Rigó, Kinga András, Tamás Hegedűs, László Cervenak

**Affiliations:** 1 Department 3rd of Internal Medicine, Semmelweis University, Budapest, Hungary; 2 Department of Pathogenetics, National Institute of Oncology, Budapest, Hungary; 3 Institute of Enzymology, Research Centre for Natural Sciences, Hungarian Academy of Sciences, Budapest, Hungary; 4 First Department of Obstetrics and Gynecology, Semmelweis University, Budapest, Hungary; 5 MTA-SE Molecular Biophysics Research Group, Hungarian Academy of Sciences, Sciences and Institute of Biophysics and Radiation Biology, Semmelweis University, Budapest, Hungary

## Abstract

Mannan-binding lectin-associated serine protease 1 (MASP-1), the most abundant enzyme of the complement lectin pathway, is able to stimulate human umbilical vein endothelial cells (HUVECs) to alter the expression of several cytokines and adhesion molecules. This study has assessed to what extent MASP-1 is able to modify the transcriptional pattern of inflammation-related (IR) genes in HUVECs. We utilized Agilent microarray to analyse the effects of recombinant MASP-1 (rMASP-1) in HUVECs, on a set of 884 IR genes. Gene Set Enrichment Analysis showed an overall activation of inflammation-related genes in response to rMASP-1. rMASP-1 treatment up- and down-regulated 19 and 11 IR genes, respectively. Most of them were previously unidentified, such as genes of chemokines (CXCL1, CXCL2, CXCL3), inflammatory receptors (TLR2, BDKRB2) and other inflammatory factors (F3, LBP). Expression of IR genes changed early, during the first 2 hours of activation. Both p38-MAPK inhibitor and NFκB inhibitor efficiently suppressed the effect of rMASP-1. We delineated 12 transcriptional factors as possible regulators of rMASP-1-induced IR genes. Our microarray-based data are in line with the hypothesis that complement lectin pathway activation, generating active MASP-1, directly regulates inflammatory processes by shifting the phenotype of endothelial cells towards a more pro-inflammatory type.

## Introduction

The complement system, a part of the innate immune system has an indispensable role in the elimination of extracellular pathogens and necrotic/apoptotic cells. The complement system can be activated through three different routes: the classical, the lectin, and the alternative pathways. Lectin pathway is specialized to recognize carbohydrate patterns on the pathogen surface, which eventually leads to the activation of the mannan-binding lectin-associated serine protease-1 (MASP-1).

Endothelial cells (ECs) form the innermost cellular lining of blood vessels and lymphatics. Endothelium, besides having important role in the regulation of several physiological functions, also participates in immunological/inflammatory processes, including leukocyte homing, antigen presentation, regulation of complement system and the clearance of immune complexes. Because of their unique anatomical localization and predisposition to inflammatory factors, the role of ECs in inflammation is crucial. We have previously demonstrated that complement MASP-1 induced human umbilical vein endothelial cells (HUVECs) acquire a pro-inflammatory phenotype, including activation of Ca^2+^-mobilization, NFκB, p38-MAPK, JNK, and CREB signaling pathways by cleaving protease-activated receptors (PARs)^1, 2^. Furthermore, MASP-1 stimulated endothelial cells are able to recruit neutrophil granulocytes via production of IL-8^1^. We also demonstrated that the expression of E-selectin adhesion molecules in HUVECs was upregulated in response to MASP-1, which resulted in increased adherence between neutrophils and endothelial cells^3^.

Inflammation is an integral, complex part of the immune-mediated response to infection, trauma, and other harmful stimuli^4, 5^. Moreover, it can also involve almost every other cell type or organ, such as liver, brain and endothelium. Activation of cells by pro-inflammatory stimuli results in a specific transcriptional output, i.e. the activation of a highly coordinated inflammation-related (IR) gene expression program^6, 7^. However, the triggering factors, the localization, as well as the cellular composition of the individual inflammatory reactions are substantially different, thus, several selective expression patterns of IR genes can be identified. These ‘foot-prints’ of transcriptional patterns are known to be distinct in the case of e.g. TNFα, lipopolysaccharide (LPS) or thrombin^8–12^, and required to an adequate response to the various provoking factors. Since the cellular effects of MASP-1 are largely unexplored, there is a rather limited knowledge on the portfolio of IR genes regulated by MASP-1.

Therefore, in our current study, we performed genome-wide gene expression profiling using the Human Genome Array to analyze the MASP-1-induced IR transcriptional pattern in HUVECs. The microarray technology provides the capacity to compare the differential expression of a large number of genes in a single assay^13^, and this is suited to confirm and expand our previous knowledge about the pro-inflammatory effects of MASP-1 on HUVECs. The possible pro-inflammatory effects of MASP-1 may contribute to the pathogenesis of inflammatory diseases, where endothelium is also involved, such as atherosclerosis, hereditary angioedema and sepsis.

## Results

### Creating a set of inflammation-related genes

We have previously demonstrated that MASP-1-induced HUVECs display a pro-inflammatory phenotype^1–3^. To focus on the expression of inflammation-related genes in response to MASP-1, we have created a novel gene set.

According to the hierarchical relationships between various functional categories in Gene Ontology Annotation (UniProt-GOA) database, there are 187 biological processes falling into the ‘Inflammatory response’ (GO:0006954) category. Based on this ‘Inflammatory response’ category, we generated a list of 642 genes, which was expanded with additional 242 IR genes from 4 commercially available Human Inflammation Kits (nCounter GX Human Inflammation Kit, nCounter Human Inflammation V2 panel, Human Inflammatory Cytokines & Receptors RT² Profiler PCR Array, Inflammatory Response & Autoimmunity PCR Array).

All the analyses with rMASP-1 and other activators were conducted with this set of 884 IR genes (Supplementary Table 1).

### Identification of rMASP-1-induced inflammation-related genes

Since primary human cell lines bear substantial inherent variability, we decided to perform analyses from multiple individual HUVEC lines to find responding genes with high fidelity. To examine the rMASP-1 effects on HUVECs, we measured the mRNA expression after rMASP-1 treatment in four independent samples. We regarded the median fold change values of the four replicates as significantly induced or suppressed by rMASP-1, if they were equal to or higher than 2, or equal to or lower than 0.5, respectively. Moreover, we analyzed whether rMASP-1 affected the IR gene set using Gene Set Enrichment Analysis (GSEA), which tests whether a given set of genes is represented higher or lower (up-regulated or down-regulated genes, respectively) in the rank list of fold changes than the average fold change of all genes. We ordered our genes according to the median log fold changes, then divided our IR gene set into up- and down-regulated genes, and found that both subsets were significantly skewed toward the top- or bottom of the rank list, respectively (Normalized enrichment score (NES) = 1.2, Nominal (NOM) p value = 0.002 for up-regulated, NES = −1.13, NOM p = 0.002 for down-regulated).

When analyzed rMASP-1 regulated IR genes, we found that rMASP-1 treatment altered 30 out of 884 (3.39%) IR genes in HUVECs, from which 19 were up- and 11 were down-regulated (Table 1). rMASP-1 was found to regulate a diverse range of IR genes, including adhesion molecules, cytokines and growth factors, and genes involved in signal transduction (Table 1).

**Table 1:** List of rMASP-1 regulated inflammation-related genes, their biological function and the effect of pathway inhibitors.

Functional classification	Gene symbol	Gene name by HUGO Gene Nomenclature Committee (HGNC)	Median of fold change by rMASP-1	NFκB inh.	p38 inh.
**Up-regulated**					
Adhesion	SELE	selectin E	3.07	x	x
	VCAM1	vascular cell adhesion molecule 1	2.08	x	x
Cytokines and growth factors	CXCL8	C-X-C motif chemokine ligand 8	5.42		x
	CXCL1	C-X-C motif chemokine ligand 1	3.47	x	x
	CXCL3	C-X-C motif chemokine ligand 3	3.32	x	x
	IL11	interleukin 11	3.17	x	x
	CXCL2	C-X-C motif chemokine ligand 2	2.66	x	x
	FGF12	fibroblast growth factor 12	2.32	x	x
	KITLG	KIT ligand	2.24		
Signaling	GHSR	growth hormone secretagogue receptor	2.78		
	NFKBID	NFKB inhibitor delta	2.48		x
	TLR2	toll like receptor 2	2.32		x
	BIRC3	baculoviral IAP repeat containing 3	2.29	x	x
	BDKRB2	bradykinin receptor B2	2.17		x
	TGFBR1	transforming growth factor beta receptor 1	2.03		x
Other	KDM6B	lysine demethylase 6B	5.52		x
	F3	coagulation factor III, tissue factor	4.09		x
	HSPB2	heat shock protein family B (small) member 2	2.98	x	x
	PRG3	proteoglycan 3, pro eosinophil major basic protein 2	2.31		x
Down-regulated*					
Cytokines and growth factors	IL2	interleukin 2	−2.22		x
Signaling	CREB3L3	cAMP responsive element binding protein 3 like 3	−2.16	x	x
	ISL1	ISL LIM homeobox 1	−2.22	x	
	CCR3	C-C motif chemokine receptor 3	−2.30		x
	FOXP3	forkhead box P3	−2.48		
	EDNRB	endothelin receptor type B	−2.90		x
	C5AR2	complement component 5a receptor 2	−2.90	x	x
	IL17RB	interleukin 17 receptor B	−2.94		x
Other	LBP	lipopolysaccharide binding protein	−2.18		x
	CHI3L1	chitinase 3 like 1	−2.30		
	PGLYRP1	peptidoglycan recognition protein 1	−2.49		x

*Median fold change (FC) of down-regulated genes are presented as −1/FC.

We have previously described that MASP-1 induced E-Selectin, IL-6 and IL-8 expression in HUVECs, which predominantly requires the involvement of p38-MAPK and NFκB signaling pathways^2^. Thus, to assess the involvement of these pathways in the regulation of IR genes, we used commercially available signaling pathway inhibitors. HUVECs were pre-incubated with the inhibitors for 30 min and then, treated with 0.6 μM rMASP-1 for 2 h. The inhibition of gene expression was considered as significant if the effect of rMASP-1 was reduced below 50% by the inhibitors. p38-MAPK inhibitor suppressed the effect of rMASP-1 more efficiently than NFκB inhibitor (83% and 40% of the rMASP-1 altered IR genes, respectively) (Table 1).

### Kinetics of inflammation-related gene expression induced by rMASP-1

**Figure 1:**
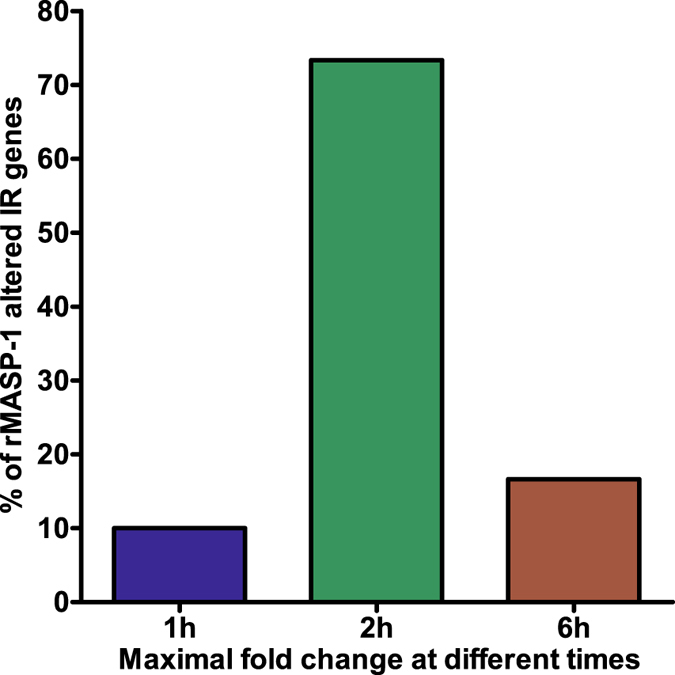
Kinetics of gene expression induced by rMASP-1. HUVECs were treated with 0.6 µM rMASP-1 for 1, 2 or 6 h then subjected to microarray analysis. Genes were categorized according to the time of maximal fold change.

As we have previously described, rMASP-1 rapidly induces IL-6, IL-8 and E-Selectin expression in endothelial cells^1, 3^. To assess the influence of rMASP-1 on the kinetics of the IR gene-expression, HUVECs were treated with 0.6 µM rMASP-1 for 1, 2 or 6 h and then microarray was performed. The expression of most rMASP-1 regulated IR genes (83%) were changed rapidly within 2 h (10% reached maximum fold change at 1 h and 73% at 2 h) (Fig. 1).

### Analysis of the transcription factors up-regulating the rMASP-1-induced inflammatory genes

**Figure 2:**
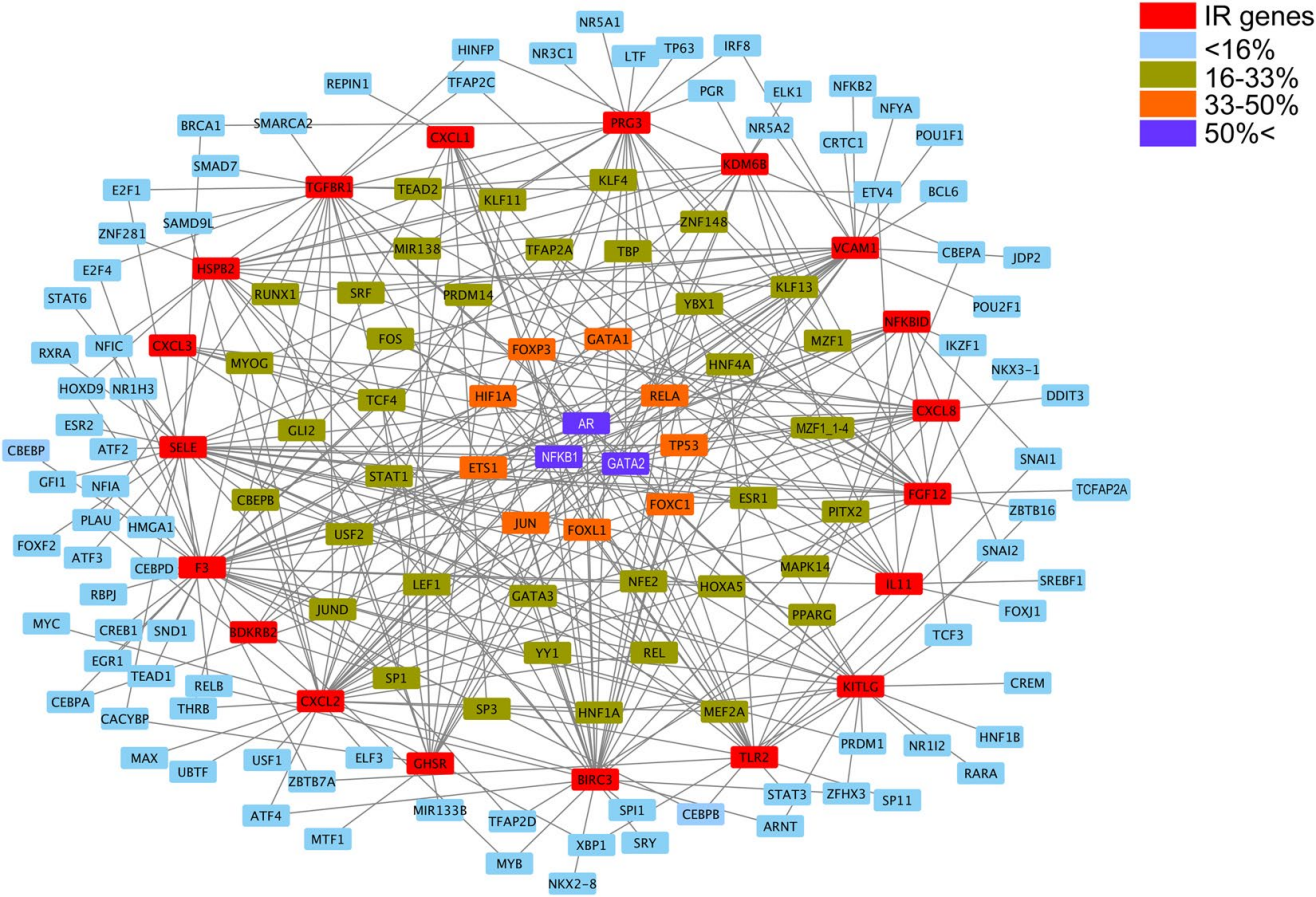
Transcription factor network of the MASP-1 induced IR genes. The graphical view of the up-regulated IR genes and the TFs which bind to their promoter regions was created with Cytoscape. Red squares represent the IR genes; blue, green, orange, and purple squares represent the TFs, which regulate less than 16%, 16–33%, 33–50%, and more than 50% of IR genes, respectively.

Although we showed that p38-MAPK pathway is one of the most important signaling pathways in MASP-1-induced adhesion molecule and cytokine production, we also demonstrated the role of other transcription factors (TF) and signaling pathways (NFκB, JNK, CREB)^1–3^. Therefore, we investigated which TFs are associated with the 19 up-regulated IR genes affected by rMASP-1. We used HTRI, TRED and Enrichr databases to identify the transcriptional regulators of each genes, and Cytoscape to analyze and visualize the collected data (Fig. 2). We found 135 TFs involved in the regulation of the up-regulated IR genes, however, most of these TFs (86) have binding site at the promoter region of less than the 16% of the rMASP-1-induced IR genes. 37 are involved in the regulation of 16–33% of IR genes, whereas 9 and 3 regulate 33–50% and more than 50% of the IR genes, respectively. We considered the last two categories of TFs as the potentially most important transcriptional regulators of rMASP-1-induced IR genes. The majority of these TFs are well-known transcriptional regulators of the inflammatory processes in endothelial cells (Table 2).

**Table 2:** The most important transcriptional factors of rMASP-1 induced IR genes.

Gene symbol	Name by HGNC	Transcriptional control event	Activated by	Binding sites in 19 rMASP-1 induced IR genes
GATA2	GATA binding protein 2	Required for preproendothelin-1 (PPET-1) and VCAM-1 gene expresson in endothelial cells^39, 40^	TNFα	14
AR	androgen receptor	Inhibition of TNFα-induced VCAM-1 expression and NF-κB activation^41^ and increasing human monocyte adhesion to vascular endothelium^42^	Testosterone (T) and 5α-dihydrotestosterone (5αDHT)	13
NFKB1	nuclear factor kappa B subunit 1	Regulates expression of E-Selectin, VCAM-1, ICAM-1, IL-6, IL-8, MCP-1 in endothelial cells^1, 3, 43^.	TNFα and Thrombin	10
ETS1	ETS proto-oncogene 1	Regulates cyclin-dependent kinase inhibitor (p21^CIP^), PAI-1, VCAM-1, and MCP-1 in endothelial cells^44^.	Ang II, PDGF-BB, and TNFα	8
FOXC1	forkhead box C1	Induces CXCR4 expression in endothelial cells, controlling CXCL12-stimulated migration^45^.	CXCL12	8
RELA	RELA proto-oncogene, NF-kB subunit	Regulates expression of E-Selectin, VCAM-1, ICAM-1, IL-6, IL-8, MCP-1 in endothelial cells^1, 3, 43^.	TNFα and Thrombin	8
FOXL1	forkhead box L1	Regulates the proliferation and differentiation of gastrointestinal epithelium^46^.	Induced during hepatic stellate cell activation	7
GATA1	GATA binding protein 1	Regulates expression of angiogenic factor with G-patch and FHA domains 1 (AGGF1) in endothelial cells^47^.	Erythropoietin (Epo)	7
TP53	tumor protein P53	Upregulates developmental endothelial locus-1 (Del-1) in endothelial cells, which inhibits leukocyte recruitment^48^.	Variety of cellular stresses	7
FOXP3	forkhead box P3	Specifies the Treg cell lineage^49^.	Activation of CD4 + T cells	6
HIF1A	hypoxia inducible factor 1 alpha subunit	Induces endothelins ET-1 and ET-2, and VEGF in endothelial cells^50^.	Hypoxia	6
JUN	Jun proto-oncogene, AP-1 transcription factor subunit	Forms AP-1, the early response transcription factor in endothelial cells^51^.	TNFα, Thrombin	6

### Comparing the effects of rMASP-1 and other pro-inflammatory factors on the IR gene expression in HUVECs

The signaling pathways, cytokines and adhesion molecules induced by the most investigated factors that provide pro-inflammatory signals to endothelial cells (such as TNFα, thrombin, histamine, or LPS) are well characterized^1, 3, 14^. Therefore, we aimed to assess whether MASP-1 can induce similar pattern of gene expression as the above-mentioned factors. The majority of the rMASP-1 altered 30 IR genes (19 up- and 11 down-regulated) were co-regulated by TNFα, thrombin, histamine, and/or LPS (10, 13, 12, 15 up-regulated IR genes and 1, 4, 2, 8 down-regulated IR genes, respectively) (Table 3

**Table 3:** Comparison of the effect of rMASP-1 and other endothelial cell activators (TNFα, thrombin, histamine, LPS).

Functional classification	rMASP-1	TNFα	TR	HA	LPS
**Up-regulated**					
Adhesion	SELE	x	x	x	x
	VCAM1	x	x	x	x
Cytokines and growth factors	CXCL8	x	x	x	x
	CXCL1	x	x	x	x
	CXCL3	x	x	x	x
	IL11		x	x	
	CXCL2	x	x	x	x
	FGF12				
	KITLG			x	x
Signaling	GHSR				x
	NFKBID		x		x
	TLR2				x
	BIRC3	x	x	x	x
	BDKRB2	x	x		
	TGFBR1		x	x	x
Other	KDM6B		x	x	x
	F3	x	x	x	x
	HSPB2				x
	PRG3	x			
	**SUM**	**10**	**13**	**12**	**15**
**Down-regulated**					
Cytokines and growth factors	IL2				x
Signaling	CREB3L3				x
	ISL1		x		
	CCR3				x
	FOXP3				x
	EDNRB	x	x	x	x
	GPR77		x	x	x
	IL17RB				
Other	LBP				
	CHI3L1		x		x
	PGLYRP1				x
	**SUM**	**1**	**4**	**2**	**8**

TNFα: tumor necrosis factor alpha, TR: thrombin, HA: histamine, LPS: lipopolysaccharide.

). Interestingly, we found considerably more rMASP-1 up-regulated genes, which were co-induced by all the other activators (7/19), than down-regulated genes (1/11).

### Verification of Microarray Analysis

To validate the analytical fidelity of microarray results, real-time quantitative PCR (qPCR) was performed for 6 target genes in HUVECs treated with rMASP-1 or TNFα (Table 4

**Table 4:** Comparisons between results from microarray analysis and qPCR.

Treatment	Method	Validation gene set						qPCR data from previous studies											
	Genes*	F3	BMP-2	TGFBR-1	EDNRB	ALOX-12	NOX-4	SELE	VCAM1	ICAM1	ICAM2	IL6	IL8	IL1A	IL1RA	MCP1	EDN1	PAI1	PLAT
rMASP-1	qPCR	5.70	1.27	1.85	−1.96	−1.51	−1.34	47.66	9.22	−1.01	−1.16	2.82	16.11	1.19	1.98	2.67	−1.03	1.38	−1.05
	MA**	5.72	1.55	1.64	−4.22	−1.69	0.95	3.56	1.50	1.11	−1.00	1.09	5.29	1.18	0.88	1.85	0.82	1.04	0.82
TNFα	qPCR	79.34	3.81	1.06	−3.36	−4.63	2.77												
	MA**	30.22	4.95	−1.66	−1.67	−1.07	−3.69												

Table 4 contains FC values calculated from qPCR and microarray data. *HGNC Gene symbol. **MA: Microarray.

). We chose 3 genes that were up-regulated, 1 that was down-regulated, whereas the expression of 2 genes did not change. Furthermore, we previously published qPCR data to evaluate cytokine and adhesion molecule expression in MASP-1 treated HUVECs, and we compared these data with those of microarrays. We found similar expression pattern in qPCR and microarray results, and Spearman test showed strong correlation (r = 0.834, p < 0.0001) between the two methods. The strong correlation between qPCR and microarray data suggests that our microarray data are reliable enough to establish conclusions for sets of genes.

## Discussion

In our earlier studies, we have demonstrated that, beside the well-established lectin pathway activating effect, complement MASP-1 has an additional pro-inflammatory role mediated by endothelial cells. HUVECs express a distinct cytokine- and adhesion molecule pattern in response to MASP-1^1, 3^. Here, we investigated a more general inflammatory role of the MASP-1, i.e. the expression of IR genes in endothelial cells by a transcriptomic approach using the Agilent Human Genome Array in HUVECs.

To exclude unnecessary or redundant information and to include all the IR genes that we found important in our previous studies regarding the pro-inflammatory effects of MASP-1 on HUVECs, we created a novel IR gene set assembled from 5 databases. It contains 884 inflammation-related genes. Our IR gene set covers the majority of genes from all inflammation-related processes: cell adhesion, cytoskeleton and extracellular matrix reorganization, arachidonic acid metabolism, production of reactive oxygen species, cytokines and hormones, as well as the inflammatory receptors and signaling pathways in the background of these processes.

GSEA analysis is a method more sensitive for the altered expression of a pathway/process specific gene set responding jointly to a stimulus than for individual genes. By this method, we found that our IR gene set is significantly regulated by rMASP-1.

Furthermore, we identified 19 and 11 IR genes that were up- or down-regulated in HUVECs, respectively. rMASP-1 altered IR genes cover the most sub-processes of inflammation, which suggests that MASP-1 has no preference for a specific part of the overall inflammatory process. This is not unexpected if we consider that e.g. the previously described neutrophil activating effects of MASP-1 via endothelial cells comprise distinct processes – chemotaxis and adhesion^1, 3^ Although thorough description of the 30 rMASP-1 regulated genes is beyond the scope of this study, we highlight some of these genes, which may add novel information to the better understanding of inflammatory processes. Based on our data, the proportion of chemokines amongst the rMASP-1 regulated genes is considerably high. CXCL1/2/3 (GRO1/2/3) are similarly neutrophil chemoattractant^15^ as CXCL8 (IL-8), which further supports our previous hypothesis that MASP-1 may preferentially recruit neutrophils via the activation of endothelial cells. The regulation of LBP and TLR2 suggests synergism between complement-based and toll-like receptor-based pattern recognition processes in order to eliminate bacteria with greater efficiency. Finally, we previously described that MASP-1 is able to cleave high molecular weight kininogen resulting bradykinin production^16, 17^. Now, we found that one of the bradykinin receptors (BDKRB2) is also up-regulated in response to MASP-1. Bradykinin-BDKRB2 system is extremely important in hereditary angioedema (HAE), a life-threatening rare disease, where the function of C1-inhibitor is diminished^18^. Low level of C1-inhibitor has been considered as a pathogenetic factor because it fails to block kallikrein^18^, the bradykinin forming enzyme. However, C1-inhibitor is also the primary inhibitor of MASP-1, therefore, our results suggest an additional pathogenetic pathway in HAE: in the absence of sufficient amount of C1-inhibitor, MASP-1 is over-activated upon microbial infection, trauma or sterile inflammation, forms bradykinin by cleaving kininogen and up-regulate BDKRB2 on endothelial cells, which together contribute to the increased permeability leading to the life-threatening edematous attacks.

We found a rapid pro-inflammatory activation of HUVECs, which appears to be associated with the alteration of 83% of rMASP-1 regulated IR genes within 2h. The kinetics of IR gene expression is concordant with our previous findings, since we described a similarly quick adhesion molecule- and cytokine production at protein level as well as an instantly elevated neutrophil chemotaxis and adhesion^1, 3^. This prompt answer leads to an efficient synchronization of the first line defense mechanisms (i.e. complement and neutrophils) against bacteria and fungi.

Activation of the NFκB and p38-MAPK signaling pathways are critical for the MASP-1-induced inflammatory stimulation of HUVECs^2^. By inhibition of these pathways, we could emphasize that both NFκB and p38-MAPK pathways directly contribute to the pro-inflammatory activation of HUVECs.

We identified 135 TFs involved in the induction of the 19 rMASP-1 up-regulated IR genes. However, only a small proportion of these TFs takes part in the regulation of a larger set of genes, which may suggest that the “promiscuous” TFs are probably more relevant in the MASP-1 signaling events than the TFs regulating only few IR genes. This hypothesis is supported by our previous study, we identified NFκB (a master transcription factor in inflammation) pathway involvement in MASP-1 signaling processes^1, 2^, and here we found that most IR genes regulated by rMASP-1 have NFκB binding site at their promoter region. Moreover, c-jun, p53 and ETS-1 were also found amongst the 12 most “promiscuous” TFs, and all of them are regulated directly by p38-MAPK^19, 20^, the most important regulator of MASP-1 signaling pathway^1, 3^. In contrast to studying TFs involved in the up-regulation of genes, assessing down-regulation is far more difficult, since there is no such database available.

Different inflammatory stimuli launch partially distinct gene sets, which results unique biological reactions^21–23^. Therefore, it is not unexpected that TNFα, histamine, thrombin, and LPS did not regulate the very same set of IR genes. On the other hand, there are several IR genes, which are regulated commonly by all activators mentioned above. rMASP-1 acted similarly to the 4 activators, i.e. their effects overlapped in some extent but we also found genes that were regulated by MASP-1 alone. This type of regulation ensures that inflammatory responses have some general features (*rubor, calor, tumor, dolor* according to the Roman encyclopedist Celsus), meanwhile each inflammatory process has some distinct features as well (e.g. histamine also induces itching, bradykinin triggers more pain receptors, LPS recruits more leukocytes than the other two inflammatory activators^1, 24, 25^). Our results suggest that MASP-1 may be an inflammation inducing enzyme, which also has some unique features based on the partially overlapping gene expression profile with other activators.

In conclusion, here we presented the very first study on the cellular effects of MASP-1 utilizing bioinformatics approaches. By GSEA method, we showed that the 884 inflammation-related genes were significantly over-represented amongst the up- and down-regulated genes, compared to the whole transcriptome. We also found several novel rMASP-1 regulated IR genes in HUVECs beyond those that were studied previously. Furthermore, the inflammation-related cellular response to rMASP-1 was found to be a rapid process, and we observed similarities as well as differences in gene expression compared to other pro-inflammatory activators. These results support our previous findings that MASP-1 may be a novel pro-inflammatory mediator, and therefore, it may be an important link between complement activation and endothelial cell induced inflammatory processes.

## Materials and Methods

### Reagents

We used recombinant catalytic fragment of human MASP-1 (CCP1-CCP2-SP, hereinafter: rMASP-1). rMASP-1 was expressed in *E. coli* ^26^, and purified according to Dobó *et al*.^27^. The rMASP-1 preparations were checked and found to be free of bacterial contaminations and could be inhibited by C1-inhibitor as described previously^1, 3, 28^. Briefly, the purity of rMASP-1 preparation exceeded 95%, checked by PAGE, the effects of rMASP-1 could be completely abrogated by C1-Inhibitor, whereas neither polymyxin B nor DNase blocked its effects. Inversely, the endothelial cell activating capacity (in NFκB translocation assay) of LPS could be decreased by polymyxin B, but not by C1-Inhibitor. Synthetic MASP-1 inhibitor SGMI-1 could also block the effects of rMASP-1. Lipoteichoic acid and fMLP did not induce NFκB nuclear translocation in HUVEC.  All other reagents were purchased from Sigma-Aldrich, unless otherwise stated. Neither rMASP-1 nor the signaling pathway inhibitors showed any sign of cytotoxicity within 24 hours in the highest concentrations utilized in the array experiments (data not shown).

### Preparation and culturing of human umbilical vein endothelial cells (HUVECs)

Cells were prepared from fresh umbilical cords obtained during normal deliveries of healthy neonates^1, 29^. HUVECs were grown in gelatin-precoated flasks (Corning® Costar®) in MCDB131 medium (Life Technologies) completed with 5% heat-inactivated fetal calf serum (FCS), 2 ng/ml human recombinant epidermal growth factor (R&D Systems), 1 ng/ml human recombinant basic fibroblast growth factor (Sigma), 0.3% Insulin Transferrin Selenium (Life Technologies), 1% Chemically Defined Lipid Concentrate (Life Technologies), 1% Glutamax (Life Technologies), 1% Penicillin-Streptomycin antibiotics (Sigma), 5 µg/ml Ascorbic acid (Sigma), 250 nM Hydrocortisone (Sigma), 10 mM Hepes (Sigma), and 7.5 U/ml Heparin. Each experiment was performed on primary HUVEC cultures from different individuals before the 4th passage. The study was conducted in conformity with the WMA Declaration of Helsinki; its protocol was approved by the Semmelweis University Institutional Review Board (permission number: TUKEB64/2008), and all participants provided their written informed consent before inclusion.

### RNA isolation and GeneChip microarray processing

To assess rMASP-1 effect on HUVECs we carried out microarrays from four distinct samples. Confluent layers of HUVECs from four individuals were cultured in 6 well plates and treated for 2 h with 0.6 µM rMASP-1. We also analyzed kinetics, effects of pathway inhibitors and other endothelial cell activators on HUVECs. For this purpose, cells were treated for 1, 2 or 6 h with 0.6 µM rMASP-1, or had been pre-incubated for 30 min with 2 µM p38-MAPK inhibitor (SB203580) or 5 µM NFκB inhibitor (Bay-11-7082) pathway inhibitors and then treated with 2 µM rMASP-1. To compare effect of MASP-1 with other endothelial cell activators, cells were treated with thrombin (300 nM), TNFα (10 ng/mL), LPS (100 ng/mL) or histamine (50 µM). Then HUVECs were lysed and stored in TRI® reagent. Total RNA purification was performed with Nucleospin^TM^ RNA XS (Macherey-Nagel), and analyzed by Agilent Bioanalyzer. The RIN of all RNA samples were above 9.

### Microarray analysis

Samples were further processed according to Agilent Two-color Microarray Based Gene Expression Analysis Low Input Quick Amp Labeling Kit protocol, and using Agilent Spike-In Kit, following the instructions provided by the manufacturer. The experimental protocol is available at: http://www.agilent.com/cs/library/usermanuals/Public/G4140-90050_GeneExpression_TwoColor_6.9.pdf

Briefly, equal amounts of Cy3-labeled (untreated) and Cy5-labeled (treated) cRNA from samples were simultaneously co-hybridized onto the arrayed oligonucleotides on the same G3 Human Gene Expression 8 × 60 K v2 Microarray (G4858A) (Agilent Technologies) slide at 65 °C for 17 h using an Agilent Gene Expression Hybridization Kit in Agilent’s SureHyb Hybridization Chambers (G2545A). The hybridized microarrays were then washed according to manufacturer’s instructions and scanned after washing using an Agilent Microarray Scanner (G2505C) at 2 µm resolution and 20-bit color-depth.

### Microarray data analysis

The scanned images were processed and analyzed by Agilent GeneSpring 14.5-GX software. We used fold change (FC) values generated by the software from the array data as the ratio of Lowess normalized, background subtracted Cy5/Cy3 signals if they passed the built-in QC analysis of the software executed according to the manufacturer’s protocol for two-color experiments. Linearization of the data was undertaken by log2 transformation.

All data are available at the Gene Expression Omnibus database at NCBI under the series accession number GSE98114.

### Validation of Microarray Data

To confirm gene expression changes measured by the microarray, quantitative gene expression was analyzed using quantitative real-time PCR (qPCR). Total RNA was isolated, purified as described above, and Promega MMLV reverse transcriptase was used for cDNA transcription. qPCR analyses were performed by LightCycler^®^ from the same samples used for microarray analysis. All primers have been designed by Primer3 (v. 0.4.0) based on NCBI database and purchased from IDT (Coralville, IA) (Table 5). Data obtained by both qPCR and microarray were normalized with values of β-actin.

**Table 5:** Primers for the analysis of mRNAs for the validation of microarray data.

	Gene	Forward	Reverse
1.	F3	5′-aggcactacaaatactgtggca-3′	5′-gcttcacatccttcacaatctcg-3′
2.	BMP2	5′-gcagcttccaccatgaagaatc-3′	5′-aaagcatcttgcatctgttctcg-3′
3.	TGFBR1	5′-tcacagagaccacagacaaagtt-3′	5′-aaagggccagtagttggaagtt-3′
4.	EDNRB	5′-catgcgaaacggtcccaatatc-3′	5′-gactcagcacagtgattccca-3′
5.	ALOX12	5′-atggtcatccagattcagcctc-3′	5′-aggtgagtgttcagcaagtgata-3′
6.	NOX4	5′-caccctgttggatgactggaa-3′	5′-actgaggtacagctggatgttg-3′
7.	SELE	5′-tcaagtgtgagcaaattgtgaac-3′	5′-attctccagaggacatacactgc-3′
8.	VCAM1	5′-tgaccttcatccctaccattga-3′	5′-gcatgtcatattcacagaactgc-3′
9.	ICAM1	5′-acagtcacctatggcaacgac-3′	5′-gtcactgtctgcagtgtctcct-3′
10.	ICAM2	5′-acagccacattcaacagcac-3′	5′-agatgtcacgaacagggacag-3′
11.	IL6	5′-ctgcaggacatgacaactcatc-3′	5′-atctgaggtgcccatgctac-3′
12.	CXCL8	5′-tcctgatttctgcagctctgt-3′	5′-tgtggtccactctcaatcactc-3′
13.	IL1A	5′-gcttcctgagcaatgtgaaatac-3′	5′-tgacttataagcacccatgtcaa-3′
14.	IL1RN	5′-gatacttgcaaggaccaaatgtc-3′	5′-gtctcatcaccagacttgacaca-3′
15.	CCL2	5′-caccaataggaagatctcagtgc-3′	5′-tgagtgttcaagtcttcggagtt-3′
16.	EDN1	5′-gagaaacccactcccagtcc-3′	5′-gatgtccaggtggcagaagt-3′
17.	SERPINE1	5′-tcaagcaagtggactttt-3′	5′-gttgaagtagagggcatt-3′
18.	PLAT	5′-gaaccacaactactgcagaaacc-3′	5′-gtgctgtgtaaaccttgcctatc-3′
19.	ACTB	5′-ggcatcctcaccctgaagta-3′	5′-ggggtgttgaaggtctcaaa-3′

### Databases, software and statistics

To create a set of the IR genes, we used the UniProt and Gene Ontology Annotation Databases^30, 31^. For the network building and analysis the Cytoscape 3.3.0 software was employed^32^. To determine the transcription factors of the genes we queried the Human Transcriptional Regulation Interactions database (HTRIdb), the Transcriptional Regulatory Element Database (TRED) and the Enrichr database^33–35^.

Gene Set Enrichment Analysis (GSEA) analysis was performed using GSEA version 2.2.3 from the Broad Institute (MIT)^36, 37^. Normalized Enrichment Scores (NES) and Nominal (NOM) p value were calculated.

We utilized the R Foundation for Statistical Computing Platform to perform Spearman correlation test^38^.

## Electronic supplementary material

Dataset 1

## References

[1] Jani PK (2014). MASP-1 induces a unique cytokine pattern in endothelial cells: a novel link between complement system and neutrophil granulocytes. PloS one.

[2] Megyeri M (2009). Complement protease MASP-1 activates human endothelial cells: PAR4 activation is a link between complement and endothelial function. J Immunol.

[3] Jani PK (2016). Complement MASP-1 enhances adhesion between endothelial cells and neutrophils by up-regulating E-selectin expression. Molecular immunology.

[4] Medzhitov R (2008). Origin and physiological roles of inflammation. Nature.

[5] Medzhitov R (2010). Inflammation 2010: new adventures of an old flame. Cell.

[6] Heinz S (2010). Simple combinations of lineage-determining transcription factors prime cis-regulatory elements required for macrophage and B cell identities. Molecular cell.

[7] Smale ST (2010). Selective transcription in response to an inflammatory stimulus. Cell.

[8] Leger AJ, Covic L, Kuliopulos A (2006). Protease-activated receptors in cardiovascular diseases. Circulation.

[9] Murakami T (2000). The gene expression profile of human umbilical vein endothelial cells stimulated by tumor necrosis factor alpha using DNA microarray analysis. Journal of atherosclerosis and thrombosis.

[10] Ukropec JA, Hollinger MK, Salva SM, Woolkalis MJ (2000). SHP2 association with VE-cadherin complexes in human endothelial cells is regulated by thrombin. The Journal of biological chemistry.

[11] Zhao B, Bowden RA, Stavchansky SA, Bowman PD (2001). Human endothelial cell response to gram-negative lipopolysaccharide assessed with cDNA microarrays. American journal of physiology. Cell physiology.

[12] Zhao B, Stavchansky SA, Bowden RA, Bowman PD (2003). Effect of interleukin-1beta and tumor necrosis factor-alpha on gene expression in human endothelial cells. American journal of physiology. Cell physiology.

[13] Schena M, Shalon D, Davis RW, Brown PO (1995). Quantitative monitoring of gene expression patterns with a complementary DNA microarray. Science.

[14] Mako V (2010). Proinflammatory activation pattern of human umbilical vein endothelial cells induced by IL-1beta, TNF-alpha, and LPS. Cytometry. Part A: the journal of the International Society for Analytical Cytology.

[15] Fox SE, Lu W, Maheshwari A, Christensen RD, Calhoun DA (2005). The effects and comparative differences of neutrophil specific chemokines on neutrophil chemotaxis of the neonate. Cytokine.

[16] Dobo J (2011). Cleavage of kininogen and subsequent bradykinin release by the complement component: mannose-binding lectin-associated serine protease (MASP)-1. PloS one.

[17] Dobo J (2014). Multiple roles of complement MASP-1 at the interface of innate immune response and coagulation. Molecular immunology.

[18] Kaplan AP, Joseph K (2010). The bradykinin-forming cascade and its role in hereditary angioedema. Annals of allergy, asthma & immunology: official publication of the American College of Allergy, Asthma, & Immunology.

[19] Cuadrado A, Nebreda AR (2010). Mechanisms and functions of p38 MAPK signalling. The Biochemical journal.

[20] Tanaka K, Oda N, Iwasaka C, Abe M, Sato Y (1998). Induction of Ets-1 in endothelial cells during reendothelialization after denuding injury. Journal of cellular physiology.

[21] Bach FH, Hancock WW, Ferran C (1997). Protective genes expressed in endothelial cells: a regulatory response to injury. Immunology today.

[22] Hawiger J (2001). Innate immunity and inflammation: a transcriptional paradigm. Immunologic research.

[23] Lentsch AB, Ward PA (2000). Regulation of inflammatory vascular damage. The Journal of pathology.

[24] Dray A, Perkins M (1993). Bradykinin and inflammatory pain. Trends in neurosciences.

[25] Shim WS, Oh U (2008). Histamine-induced itch and its relationship with pain. Molecular pain.

[26] Ambrus G (2003). Natural substrates and inhibitors of mannan-binding lectin-associated serine protease-1 and -2: a study on recombinant catalytic fragments. J Immunol.

[27] Dobo J (2008). Purification, crystallization and preliminary X-ray analysis of human mannose-binding lectin-associated serine protease-1 (MASP-1) catalytic region. Acta crystallographica. Section F, Structural biology and crystallization communications.

[28] Megyeri M (2014). Serum MASP-1 in complex with MBL activates endothelial cells. Molecular immunology.

[29] Oroszlan M (2006). Proinflammatory changes in human umbilical cord vein endothelial cells can be induced neither by native nor by modified CRP. International immunology.

[30] Gene Ontology Consortium: going forward. *Nucleic acids research* **43**, D1049–1056 (2015). 10.1093/nar/gku1179 PMC4383973 25428369

[31] UniProt: a hub for protein information. *Nucleic acids research* **43**, D204–212 (2015). 10.1093/nar/gku989 PMC4384041 25348405

[32] Shannon P (2003). Cytoscape: a software environment for integrated models of biomolecular interaction networks. Genome research.

[33] Bovolenta LA, Acencio ML, Lemke N (2012). HTRIdb: an open-access database for experimentally verified human transcriptional regulation interactions. BMC genomics.

[34] Chen EY (2013). Enrichr: interactive and collaborative HTML5 gene list enrichment analysis tool. BMC bioinformatics.

[35] Zhao F, Xuan Z, Liu L, Zhang MQ (2005). TRED: a Transcriptional Regulatory Element Database and a platform for in silico gene regulation studies. Nucleic acids research.

[36] Mootha VK (2003). PGC-1alpha-responsive genes involved in oxidative phosphorylation are coordinately downregulated in human diabetes. Nature genetics.

[37] Subramanian A (2005). Gene set enrichment analysis: a knowledge-based approach for interpreting genome-wide expression profiles. Proceedings of the National Academy of Sciences of the United States of America.

[38] R: A language and environment for statistical computing. (R Foundation for Statistical Computing, Vienna, Austria, 2015).

[39] Dorfman DM, Wilson DB, Bruns GA, Orkin SH (1992). Human transcription factor GATA-2. Evidence for regulation of preproendothelin-1 gene expression in endothelial cells. The Journal of biological chemistry.

[40] Umetani M (2001). Function of GATA transcription factors in induction of endothelial vascular cell adhesion molecule-1 by tumor necrosis factor-alpha. Arteriosclerosis, thrombosis, and vascular biology.

[41] Hatakeyama H (2002). Testosterone inhibits tumor necrosis factor-alpha-induced vascular cell adhesion molecule-1 expression in human aortic endothelial cells. FEBS letters.

[42] McCrohon JA, Jessup W, Handelsman DJ, Celermajer DS (1999). Androgen exposure increases human monocyte adhesion to vascular endothelium and endothelial cell expression of vascular cell adhesion molecule-1. Circulation.

[43] Hoesel B, Schmid JA (2013). The complexity of NF-kappaB signaling in inflammation and cancer. Molecular cancer.

[44] Zhan Y (2005). Ets-1 is a critical regulator of Ang II-mediated vascular inflammation and remodeling. The Journal of clinical investigation.

[45] Hayashi H, Kume T (2008). Forkhead transcription factors regulate expression of the chemokine receptor CXCR4 in endothelial cells and CXCL12-induced cell migration. Biochemical and biophysical research communications.

[46] Kaestner KH, Silberg DG, Traber PG, Schutz G (1997). The mesenchymal winged helix transcription factor Fkh6 is required for the control of gastrointestinal proliferation and differentiation. Genes & development.

[47] Fan C (2009). Novel roles of GATA1 in regulation of angiogenic factor AGGF1 and endothelial cell function. The Journal of biological chemistry.

[48] Kim H (2013). p53 regulates the transcription of the anti-inflammatory molecule developmental endothelial locus-1 (Del-1). Oncotarget.

[49] Josefowicz SZ, Lu LF, Rudensky AY (2012). Regulatory T cells: mechanisms of differentiation and function. Annual review of immunology.

[50] Palazon A, Goldrath AW, Nizet V, Johnson RS (2014). HIF transcription factors, inflammation, and immunity. Immunity.

[51] Jia J (2016). AP-1 transcription factor mediates VEGF-induced endothelial cell migration and proliferation. Microvascular research.

