# Supplementary material for: Transcriptome analysis of inflammation-related gene expression in endothelial cells activated by complement MASP-1
Source: Sci Rep. 2017 Sep 5;7:10462. doi: 10.1038/s41598-017-09058-8 (PMC5585174; doi:10.1038/s41598-017-09058-8)
Supplement: Supplementary file 1 — Dataset 1 [file 41598_2017_9058_MOESM1_ESM.doc]

**Transcriptome analysis of inflammation-related gene expression in endothelial cells activated by complement MASP-1**

**Endre Schwaner, Zsuzsanna Németh, Péter K. Jani, Erika Kajdácsi, Márta L. Debreczeni, Zoltán Doleschall, József Dobó, Péter Gál, János Rigó Jr., Kinga András, Tamás Hegedűs & László Cervenak^*^**

*To whom correspondence should be addressed: Dr. László Cervenak, 3rd Department of Internal Medicine, Semmelweis University, Budapest, Hungary, 1125 Budapest, Kútvölgyi street 4., Hungary. Phone: +36 1 325-1494, Fax: +36 1 212-9351, E-mail address: [cervenak.laszlo@med.semmelweis-univ.hu](mailto:cervenak.laszlo@med.semmelweis-univ.hu)

**Supplementary Table 1. The list of 884 inflammation-related (IR) genes**

|  | **IR gene symbol** | **IR gene name by HGNC** |
| --- | --- | --- |
| 1. | **A2M** | alpha-2-macroglobulin |
| 2. | **ABCF1** | ATP binding cassette subfamily F member 1 |
| 3. | **ABR** | active BCR-related |
| 4. | **ACE2** | angiotensin I converting enzyme 2 |
| 5. | **ACKR1** | atypical chemokine receptor 1 (Duffy blood group) |
| 6. | **ACKR2** | atypical chemokine receptor 2 |
| 7. | **ACKR4** | atypical chemokine receptor 4 |
| 8. | **ACOD1** | aconitate decarboxylase 1 |
| 9. | **ACP5** | acid phosphatase 5, tartrate resistant |
| 10. | **ACVR1** | activin A receptor type 1 |
| 11. | **ADA** | adenosine deaminase |
| 12. | **ADAM8** | ADAM metallopeptidase domain 8 |
| 13. | **ADAMTS12** | ADAM metallopeptidase with thrombospondin type 1 motif 12 |
| 14. | **ADCYAP1** | adenylate cyclase activating polypeptide 1 |
| 15. | **ADGRE2** | adhesion G protein-coupled receptor E2 |
| 16. | **ADGRE5** | adhesion G protein-coupled receptor E5 |
| 17. | **ADIPOQ** | adiponectin, C1Q and collagen domain containing |
| 18. | **ADORA1** | adenosine A1 receptor |
| 19. | **ADORA2A** | adenosine A2a receptor |
| 20. | **ADORA2B** | adenosine A2b receptor |
| 21. | **ADRA2A** | adrenoceptor alpha 2A |
| 22. | **ADRB2** | adrenoceptor beta 2 |
| 23. | **AFAP1L2** | actin filament associated protein 1 like 2 |
| 24. | **AGER** | advanced glycosylation end-product specific receptor |
| 25. | **AGT** | angiotensinogen |
| 26. | **AGTR1** | angiotensin II receptor type 1 |
| 27. | **AGTR2** | angiotensin II receptor type 2 |
| 28. | **AHCY** | adenosylhomocysteinase |
| 29. | **AHSG** | alpha 2-HS glycoprotein |
| 30. | **AIF1** | allograft inflammatory factor 1 |
| 31. | **AIM2** | absent in melanoma 2 |
| 32. | **AIMP1** | aminoacyl tRNA synthetase complex interacting multifunctional protein 1 |
| 33. | **AK7** | adenylate kinase 7 |
| 34. | **AKT1** | AKT serine/threonine kinase 1 |
| 35. | **ALOX12** | arachidonate 12-lipoxygenase, 12S type |
| 36. | **ALOX15** | arachidonate 15-lipoxygenase |
| 37. | **ALOX5** | arachidonate 5-lipoxygenase |
| 38. | **ANO6** | anoctamin 6 |
| 39. | **ANXA1** | annexin A1 |
| 40. | **AOAH** | acyloxyacyl hydrolase |
| 41. | **AOC3** | amine oxidase, copper containing 3 |
| 42. | **AOX1** | aldehyde oxidase 1 |
| 43. | **APCS** | amyloid P component, serum |
| 44. | **APOA1** | apolipoprotein A1 |
| 45. | **APOA2** | apolipoprotein A2 |
| 46. | **APOC3** | apolipoprotein C3 |
| 47. | **APOD** | apolipoprotein D |
| 48. | **APOE** | apolipoprotein E |
| 49. | **APOL2** | apolipoprotein L2 |
| 50. | **APOL3** | apolipoprotein L3 |
| 51. | **AREG** | amphiregulin |
| 52. | **ARG1** | arginase 1 |
| 53. | **ASH1L** | ASH1 like histone lysine methyltransferase |
| 54. | **ASS1** | argininosuccinate synthase 1 |
| 55. | **ATF2** | activating transcription factor 2 |
| 56. | **ATRN** | attractin |
| 57. | **AXL** | AXL receptor tyrosine kinase |
| 58. | **AZU1** | azurocidin 1 |
| 59. | **B4GALT1** | beta-1,4-galactosyltransferase 1 |
| 60. | **BCL2L1** | BCL2 like 1 |
| 61. | **BCL6** | B-cell CLL/lymphoma 6 |
| 62. | **BCR** | BCR, RhoGEF and GTPase activating protein |
| 63. | **BDKRB1** | bradykinin receptor B1 |
| 64. | **BDKRB2** | bradykinin receptor B2 |
| 65. | **BIRC2** | baculoviral IAP repeat containing 2 |
| 66. | **BIRC3** | baculoviral IAP repeat containing 3 |
| 67. | **BLNK** | B-cell linker |
| 68. | **BMP1** | bone morphogenetic protein 1 |
| 69. | **BMP2** | bone morphogenetic protein 2 |
| 70. | **BMP3** | bone morphogenetic protein 3 |
| 71. | **BMP6** | bone morphogenetic protein 6 |
| 72. | **BMP7** | bone morphogenetic protein 7 |
| 73. | **BMPR1B** | bone morphogenetic protein receptor type 1B |
| 74. | **BRD4** | bromodomain containing 4 |
| 75. | **C1QA** | complement C1q A chain |
| 76. | **C1QB** | complement C1q B chain |
| 77. | **C1QBP** | complement C1q binding protein |
| 78. | **C1QTNF12** | C1q and tumor necrosis factor related protein 12 |
| 79. | **C1QTNF3** | C1q and tumor necrosis factor related protein 3 |
| 80. | **C1R** | complement C1r |
| 81. | **C1S** | complement C1s |
| 82. | **C2** | complement C2 |
| 83. | **C3** | complement C3 |
| 84. | **C3AR1** | complement C3a receptor 1 |
| 85. | **C4B** | complement C4B (Chido blood group) |
| 86. | **C4BPA** | complement component 4 binding protein alpha |
| 87. | **C4BPB** | complement component 4 binding protein beta |
| 88. | **C5** | complement C5 |
| 89. | **C5AR1** | complement C5a receptor 1 |
| 90. | **C5AR2** | complement component 5a receptor 2 |
| 91. | **C6** | complement C6 |
| 92. | **C7** | complement C7 |
| 93. | **C8A** | complement C8 alpha chain |
| 94. | **C8B** | complement C8 beta chain |
| 95. | **C8G** | complement C8 gamma chain |
| 96. | **C9** | complement C9 |
| 97. | **CALCA** | calcitonin related polypeptide alpha |
| 98. | **CALCRL** | calcitonin receptor like receptor |
| 99. | **CAMK1D** | calcium/calmodulin dependent protein kinase ID |
| 100. | **CAMK4** | calcium/calmodulin dependent protein kinase IV |
| 101. | **CAMP** | cathelicidin antimicrobial peptide |
| 102. | **CARD18** | caspase recruitment domain family member 18 |
| 103. | **CASP4** | caspase 4 |
| 104. | **CASP5** | caspase 5 |
| 105. | **CAST** | calpastatin |
| 106. | **CCL1** | C-C motif chemokine ligand 1 |
| 107. | **CCL11** | C-C motif chemokine ligand 11 |
| 108. | **CCL13** | C-C motif chemokine ligand 13 |
| 109. | **CCL14** | C-C motif chemokine ligand 14 |
| 110. | **CCL15** | C-C motif chemokine ligand 15 |
| 111. | **CCL16** | C-C motif chemokine ligand 16 |
| 112. | **CCL17** | C-C motif chemokine ligand 17 |
| 113. | **CCL18** | C-C motif chemokine ligand 18 |
| 114. | **CCL19** | C-C motif chemokine ligand 19 |
| 115. | **CCL2** | C-C motif chemokine ligand 2 |
| 116. | **CCL20** | C-C motif chemokine ligand 20 |
| 117. | **CCL21** | C-C motif chemokine ligand 21 |
| 118. | **CCL22** | C-C motif chemokine ligand 22 |
| 119. | **CCL23** | C-C motif chemokine ligand 23 |
| 120. | **CCL24** | C-C motif chemokine ligand 24 |
| 121. | **CCL25** | C-C motif chemokine ligand 25 |
| 122. | **CCL26** | C-C motif chemokine ligand 26 |
| 123. | **CCL27** | C-C motif chemokine ligand 27 |
| 124. | **CCL28** | C-C motif chemokine ligand 28 |
| 125. | **CCL3** | C-C motif chemokine ligand 3 |
| 126. | **CCL4** | C-C motif chemokine ligand 4 |
| 127. | **CCL5** | C-C motif chemokine ligand 5 |
| 128. | **CCL7** | C-C motif chemokine ligand 7 |
| 129. | **CCL8** | C-C motif chemokine ligand 8 |
| 130. | **CCR1** | C-C motif chemokine receptor 1 |
| 131. | **CCR10** | C-C motif chemokine receptor 10 |
| 132. | **CCR2** | C-C motif chemokine receptor 2 |
| 133. | **CCR3** | C-C motif chemokine receptor 3 |
| 134. | **CCR4** | C-C motif chemokine receptor 4 |
| 135. | **CCR5** | C-C motif chemokine receptor 5 (gene/pseudogene) |
| 136. | **CCR6** | C-C motif chemokine receptor 6 |
| 137. | **CCR7** | C-C motif chemokine receptor 7 |
| 138. | **CCR8** | C-C motif chemokine receptor 8 |
| 139. | **CCR9** | C-C motif chemokine receptor 9 |
| 140. | **CCRL2** | C-C motif chemokine receptor like 2 |
| 141. | **CD14** | CD14 molecule |
| 142. | **CD163** | CD163 molecule |
| 143. | **CD180** | CD180 molecule |
| 144. | **CD27** | CD27 molecule |
| 145. | **CD276** | CD276 molecule |
| 146. | **CD28** | CD28 molecule |
| 147. | **CD4** | CD4 molecule |
| 148. | **CD40** | CD40 molecule |
| 149. | **CD40LG** | CD40 ligand |
| 150. | **CD44** | CD44 molecule (Indian blood group) |
| 151. | **CD46** | CD46 molecule |
| 152. | **CD47** | CD47 molecule |
| 153. | **CD55** | CD55 molecule (Cromer blood group) |
| 154. | **CD59** | CD59 molecule |
| 155. | **CD6** | CD6 molecule |
| 156. | **CD70** | CD70 molecule |
| 157. | **CD74** | CD74 molecule |
| 158. | **CD86** | CD86 molecule |
| 159. | **CDC42** | cell division cycle 42 |
| 160. | **CDK19** | cyclin dependent kinase 19 |
| 161. | **CDO1** | cysteine dioxygenase type 1 |
| 162. | **CEBPB** | CCAAT/enhancer binding protein beta |
| 163. | **CELA1** | chymotrypsin like elastase family member 1 |
| 164. | **CFB** | complement factor B |
| 165. | **CFD** | complement factor D |
| 166. | **CFH** | complement factor H |
| 167. | **CFI** | complement factor I |
| 168. | **CFL1** | cofilin 1 |
| 169. | **CFP** | complement factor properdin |
| 170. | **CHI3L1** | chitinase 3 like 1 |
| 171. | **CHIA** | chitinase, acidic |
| 172. | **CHID1** | chitinase domain containing 1 |
| 173. | **CHRFAM7A** | CHRNA7 (exons 5-10) and FAM7A (exons A-E) fusion |
| 174. | **CHST1** | carbohydrate sulfotransferase 1 |
| 175. | **CHST2** | carbohydrate sulfotransferase 2 |
| 176. | **CHST4** | carbohydrate sulfotransferase 4 |
| 177. | **CHUK** | conserved helix-loop-helix ubiquitous kinase |
| 178. | **CKLF** | chemokine like factor |
| 179. | **CLC** | Charcot-Leyden crystal galectin |
| 180. | **CLEC7A** | C-type lectin domain family 7 member A |
| 181. | **CLOCK** | clock circadian regulator |
| 182. | **CMA1** | chymase 1 |
| 183. | **CMTM1** | CKLF like MARVEL transmembrane domain containing 1 |
| 184. | **CMTM2** | CKLF like MARVEL transmembrane domain containing 2 |
| 185. | **CNR1** | cannabinoid receptor 1 |
| 186. | **CNR2** | cannabinoid receptor 2 |
| 187. | **CNTFR** | ciliary neurotrophic factor receptor |
| 188. | **CR1** | complement C3b/C4b receptor 1 (Knops blood group) |
| 189. | **CREB1** | cAMP responsive element binding protein 1 |
| 190. | **CREB3L3** | cAMP responsive element binding protein 3 like 3 |
| 191. | **CRH** | corticotropin releasing hormone |
| 192. | **CRHBP** | corticotropin releasing hormone binding protein |
| 193. | **CRP** | C-reactive protein |
| 194. | **CSF1** | colony stimulating factor 1 |
| 195. | **CSF1R** | colony stimulating factor 1 receptor |
| 196. | **CSF2** | colony stimulating factor 2 |
| 197. | **CSF2RA** | colony stimulating factor 2 receptor alpha subunit |
| 198. | **CSF2RB** | colony stimulating factor 2 receptor beta common subunit |
| 199. | **CSF3** | colony stimulating factor 3 |
| 200. | **CSF3R** | colony stimulating factor 3 receptor |
| 201. | **CTF1** | cardiotrophin 1 |
| 202. | **CTNNBIP1** | catenin beta interacting protein 1 |
| 203. | **CTSS** | cathepsin S |
| 204. | **CUEDC2** | CUE domain containing 2 |
| 205. | **CX3CL1** | C-X3-C motif chemokine ligand 1 |
| 206. | **CX3CR1** | C-X3-C motif chemokine receptor 1 |
| 207. | **CXCL1** | C-X-C motif chemokine ligand 1 |
| 208. | **CXCL10** | C-X-C motif chemokine ligand 10 |
| 209. | **CXCL11** | C-X-C motif chemokine ligand 11 |
| 210. | **CXCL12** | C-X-C motif chemokine ligand 12 |
| 211. | **CXCL13** | C-X-C motif chemokine ligand 13 |
| 212. | **CXCL14** | C-X-C motif chemokine ligand 14 |
| 213. | **CXCL16** | C-X-C motif chemokine ligand 16 |
| 214. | **CXCL2** | C-X-C motif chemokine ligand 2 |
| 215. | **CXCL3** | C-X-C motif chemokine ligand 3 |
| 216. | **CXCL5** | C-X-C motif chemokine ligand 5 |
| 217. | **CXCL6** | C-X-C motif chemokine ligand 6 |
| 218. | **CXCL8** | C-X-C motif chemokine ligand 8 |
| 219. | **CXCL9** | C-X-C motif chemokine ligand 9 |
| 220. | **CXCR1** | C-X-C motif chemokine receptor 1 |
| 221. | **CXCR2** | C-X-C motif chemokine receptor 2 |
| 222. | **CXCR3** | C-X-C motif chemokine receptor 3 |
| 223. | **CXCR4** | C-X-C motif chemokine receptor 4 |
| 224. | **CXCR5** | C-X-C motif chemokine receptor 5 |
| 225. | **CXCR6** | C-X-C motif chemokine receptor 6 |
| 226. | **CYBA** | cytochrome b-245 alpha chain |
| 227. | **CYBB** | cytochrome b-245 beta chain |
| 228. | **CYP19A1** | cytochrome P450 family 19 subfamily A member 1 |
| 229. | **CYP26B1** | cytochrome P450 family 26 subfamily B member 1 |
| 230. | **CYP4F11** | cytochrome P450 family 4 subfamily F member 11 |
| 231. | **CYSLTR1** | cysteinyl leukotriene receptor 1 |
| 232. | **CYSLTR2** | cysteinyl leukotriene receptor 2 |
| 233. | **DAB2IP** | DAB2 interacting protein |
| 234. | **DAXX** | death domain associated protein |
| 235. | **DDIT3** | DNA damage inducible transcript 3 |
| 236. | **DEFB1** | defensin beta 1 |
| 237. | **DOCK2** | dedicator of cytokinesis 2 |
| 238. | **DUOXA1** | dual oxidase maturation factor 1 |
| 239. | **DUOXA2** | dual oxidase maturation factor 2 |
| 240. | **DUSP10** | dual specificity phosphatase 10 |
| 241. | **EBI3** | Epstein-Barr virus induced 3 |
| 242. | **ECM1** | extracellular matrix protein 1 |
| 243. | **EDA** | ectodysplasin A |
| 244. | **EDNRA** | endothelin receptor type A |
| 245. | **EDNRB** | endothelin receptor type B |
| 246. | **EIF2AK1** | eukaryotic translation initiation factor 2 alpha kinase 1 |
| 247. | **ELANE** | elastase, neutrophil expressed |
| 248. | **ELF3** | E74 like ETS transcription factor 3 |
| 249. | **ELK1** | ELK1, ETS transcription factor |
| 250. | **EPHX2** | epoxide hydrolase 2 |
| 251. | **EPO** | erythropoietin |
| 252. | **EPOR** | erythropoietin receptor |
| 253. | **ERBB2** | erb-b2 receptor tyrosine kinase 2 |
| 254. | **ERBIN** | erbb2 interacting protein |
| 255. | **ETS1** | ETS proto-oncogene 1, transcription factor |
| 256. | **F11R** | F11 receptor |
| 257. | **F12** | coagulation factor XII |
| 258. | **F2** | coagulation factor II, thrombin |
| 259. | **F2R** | coagulation factor II thrombin receptor |
| 260. | **F2RL1** | F2R like trypsin receptor 1 |
| 261. | **F3** | coagulation factor III, tissue factor |
| 262. | **F8** | coagulation factor VIII |
| 263. | **FABP4** | fatty acid binding protein 4 |
| 264. | **FAS** | Fas cell surface death receptor |
| 265. | **FASLG** | Fas ligand |
| 266. | **FCER1A** | Fc fragment of IgE receptor Ia |
| 267. | **FCER1G** | Fc fragment of IgE receptor Ig |
| 268. | **FEM1A** | fem-1 homolog A |
| 269. | **FFAR2** | free fatty acid receptor 2 |
| 270. | **FFAR3** | free fatty acid receptor 3 |
| 271. | **FFAR4** | free fatty acid receptor 4 |
| 272. | **FGA** | fibrinogen alpha chain |
| 273. | **FGF1** | fibroblast growth factor 1 |
| 274. | **FGF10** | fibroblast growth factor 10 |
| 275. | **FGF12** | fibroblast growth factor 12 |
| 276. | **FGF2** | fibroblast growth factor 2 |
| 277. | **FGF7** | fibroblast growth factor 7 |
| 278. | **FLT1** | fms related tyrosine kinase 1 |
| 279. | **FLT3LG** | fms related tyrosine kinase 3 ligand |
| 280. | **FN1** | fibronectin 1 |
| 281. | **FOLR2** | folate receptor beta |
| 282. | **FOS** | Fos proto-oncogene, AP-1 transcription factor subunit |
| 283. | **FOXP1** | forkhead box P1 |
| 284. | **FOXP3** | forkhead box P3 |
| 285. | **FPR1** | formyl peptide receptor 1 |
| 286. | **FPR2** | formyl peptide receptor 2 |
| 287. | **FXYD2** | FXYD domain containing ion transport regulator 2 |
| 288. | **GAL** | galanin and GMAP prepropeptide |
| 289. | **GATA3** | GATA binding protein 3 |
| 290. | **GBA** | glucosylceramidase beta |
| 291. | **GBP5** | guanylate binding protein 5 |
| 292. | **GDF2** | growth differentiation factor 2 |
| 293. | **GDF3** | growth differentiation factor 3 |
| 294. | **GDF5** | growth differentiation factor 5 |
| 295. | **GDF6** | growth differentiation factor 6 |
| 296. | **GDF9** | growth differentiation factor 9 |
| 297. | **GFRA1** | GDNF family receptor alpha 1 |
| 298. | **GFRA2** | GDNF family receptor alpha 2 |
| 299. | **GGT1** | gamma-glutamyltransferase 1 |
| 300. | **GGT5** | gamma-glutamyltransferase 5 |
| 301. | **GHR** | growth hormone receptor |
| 302. | **GHRL** | ghrelin and obestatin prepropeptide |
| 303. | **GHSR** | growth hormone secretagogue receptor |
| 304. | **GJA1** | gap junction protein alpha 1 |
| 305. | **GLMN** | glomulin, FKBP associated protein |
| 306. | **GNAQ** | G protein subunit alpha q |
| 307. | **GNAS** | GNAS complex locus |
| 308. | **GNB1** | G protein subunit beta 1 |
| 309. | **GNGT1** | G protein subunit gamma transducin 1 |
| 310. | **GPER1** | G protein-coupled estrogen receptor 1 |
| 311. | **GPI** | glucose-6-phosphate isomerase |
| 312. | **GPR68** | G protein-coupled receptor 68 |
| 313. | **GPRC5B** | G protein-coupled receptor class C group 5 member B |
| 314. | **GPX1** | glutathione peroxidase 1 |
| 315. | **GRB2** | growth factor receptor bound protein 2 |
| 316. | **GREM1** | gremlin 1, DAN family BMP antagonist |
| 317. | **GREM2** | gremlin 2, DAN family BMP antagonist |
| 318. | **GRN** | granulin precursor |
| 319. | **GSTP1** | glutathione S-transferase pi 1 |
| 320. | **HAMP** | hepcidin antimicrobial peptide |
| 321. | **HCK** | HCK proto-oncogene, Src family tyrosine kinase |
| 322. | **HDAC4** | histone deacetylase 4 |
| 323. | **HDAC5** | histone deacetylase 5 |
| 324. | **HDAC7** | histone deacetylase 7 |
| 325. | **HDAC9** | histone deacetylase 9 |
| 326. | **HFE** | hemochromatosis |
| 327. | **HIF1A** | hypoxia inducible factor 1 alpha subunit |
| 328. | **HIST1H2BA** | histone cluster 1 H2B family member a |
| 329. | **HLA-DRA** | major histocompatibility complex, class II, DR alpha |
| 330. | **HLA-DRB1** | major histocompatibility complex, class II, DR beta 1 |
| 331. | **HMGB1** | high mobility group box 1 |
| 332. | **HMGB2** | high mobility group box 2 |
| 333. | **HMGN1** | high mobility group nucleosome binding domain 1 |
| 334. | **HMOX1** | heme oxygenase 1 |
| 335. | **HNRNPA0** | heterogeneous nuclear ribonucleoprotein A0 |
| 336. | **HNRNPK** | heterogeneous nuclear ribonucleoprotein K |
| 337. | **HP** | haptoglobin |
| 338. | **HRAS** | HRas proto-oncogene, GTPase |
| 339. | **HRH1** | histamine receptor H1 |
| 340. | **HRH4** | histamine receptor H4 |
| 341. | **HSH2D** | hematopoietic SH2 domain containing |
| 342. | **HSPB1** | heat shock protein family B (small) member 1 |
| 343. | **HSPB2** | heat shock protein family B (small) member 2 |
| 344. | **HSPD1** | heat shock protein family D (Hsp60) member 1 |
| 345. | **HYAL1** | hyaluronoglucosaminidase 1 |
| 346. | **HYAL2** | hyaluronoglucosaminidase 2 |
| 347. | **HYAL3** | hyaluronoglucosaminidase 3 |
| 348. | **ICAM1** | intercellular adhesion molecule 1 |
| 349. | **IDO1** | indoleamine 2,3-dioxygenase 1 |
| 350. | **IER3** | immediate early response 3 |
| 351. | **IFI16** | interferon gamma inducible protein 16 |
| 352. | **IFI44** | interferon induced protein 44 |
| 353. | **IFIT1** | interferon induced protein with tetratricopeptide repeats 1 |
| 354. | **IFIT2** | interferon induced protein with tetratricopeptide repeats 2 |
| 355. | **IFIT3** | interferon induced protein with tetratricopeptide repeats 3 |
| 356. | **IFNA14** | interferon alpha 14 |
| 357. | **IFNA2** | interferon alpha 2 |
| 358. | **IFNA4** | interferon alpha 4 |
| 359. | **IFNA8** | interferon alpha 8 |
| 360. | **IFNAR1** | interferon alpha and beta receptor subunit 1 |
| 361. | **IFNAR2** | interferon alpha and beta receptor subunit 2 |
| 362. | **IFNB1** | interferon beta 1 |
| 363. | **IFNE** | interferon epsilon |
| 364. | **IFNG** | interferon gamma |
| 365. | **IFNGR1** | interferon gamma receptor 1 |
| 366. | **IFNGR2** | interferon gamma receptor 2 |
| 367. | **IFNK** | interferon kappa |
| 368. | **IFNL1** | interferon lambda 1 |
| 369. | **IFNLR1** | interferon lambda receptor 1 |
| 370. | **IFNW1** | interferon omega 1 |
| 371. | **IGFBP4** | insulin like growth factor binding protein 4 |
| 372. | **IK** | IK cytokine, down-regulator of HLA II |
| 373. | **IKBKB** | inhibitor of nuclear factor kappa B kinase subunit beta |
| 374. | **IKBKG** | inhibitor of nuclear factor kappa B kinase subunit gamma |
| 375. | **IL10** | interleukin 10 |
| 376. | **IL10RA** | interleukin 10 receptor subunit alpha |
| 377. | **IL10RB** | interleukin 10 receptor subunit beta |
| 378. | **IL11** | interleukin 11 |
| 379. | **IL11RA** | interleukin 11 receptor subunit alpha |
| 380. | **IL12A** | interleukin 12A |
| 381. | **IL12B** | interleukin 12B |
| 382. | **IL12RB1** | interleukin 12 receptor subunit beta 1 |
| 383. | **IL12RB2** | interleukin 12 receptor subunit beta 2 |
| 384. | **IL13** | interleukin 13 |
| 385. | **IL13RA1** | interleukin 13 receptor subunit alpha 1 |
| 386. | **IL13RA2** | interleukin 13 receptor subunit alpha 2 |
| 387. | **IL15** | interleukin 15 |
| 388. | **IL15RA** | interleukin 15 receptor subunit alpha |
| 389. | **IL16** | interleukin 16 |
| 390. | **IL17A** | interleukin 17A |
| 391. | **IL17B** | interleukin 17B |
| 392. | **IL17C** | interleukin 17C |
| 393. | **IL17D** | interleukin 17D |
| 394. | **IL17F** | interleukin 17F |
| 395. | **IL17RA** | interleukin 17 receptor A |
| 396. | **IL17RB** | interleukin 17 receptor B |
| 397. | **IL17RC** | interleukin 17 receptor C |
| 398. | **IL17RE** | interleukin 17 receptor E |
| 399. | **IL18** | interleukin 18 |
| 400. | **IL18R1** | interleukin 18 receptor 1 |
| 401. | **IL18RAP** | interleukin 18 receptor accessory protein |
| 402. | **IL19** | interleukin 19 |
| 403. | **IL1A** | interleukin 1 alpha |
| 404. | **IL1B** | interleukin 1 beta |
| 405. | **IL1F10** | interleukin 1 family member 10 (theta) |
| 406. | **IL1R1** | interleukin 1 receptor type 1 |
| 407. | **IL1R2** | interleukin 1 receptor type 2 |
| 408. | **IL1RAP** | interleukin 1 receptor accessory protein |
| 409. | **IL1RAPL2** | interleukin 1 receptor accessory protein like 2 |
| 410. | **IL1RL1** | interleukin 1 receptor like 1 |
| 411. | **IL1RL2** | interleukin 1 receptor like 2 |
| 412. | **IL1RN** | interleukin 1 receptor antagonist |
| 413. | **IL2** | interleukin 2 |
| 414. | **IL20** | interleukin 20 |
| 415. | **IL20RA** | interleukin 20 receptor subunit alpha |
| 416. | **IL20RB** | interleukin 20 receptor subunit beta |
| 417. | **IL21** | interleukin 21 |
| 418. | **IL21R** | interleukin 21 receptor |
| 419. | **IL22** | interleukin 22 |
| 420. | **IL22RA1** | interleukin 22 receptor subunit alpha 1 |
| 421. | **IL22RA2** | interleukin 22 receptor subunit alpha 2 |
| 422. | **IL23A** | interleukin 23 subunit alpha |
| 423. | **IL23R** | interleukin 23 receptor |
| 424. | **IL24** | interleukin 24 |
| 425. | **IL25** | interleukin 25 |
| 426. | **IL26** | interleukin 26 |
| 427. | **IL27** | interleukin 27 |
| 428. | **IL2RA** | interleukin 2 receptor subunit alpha |
| 429. | **IL2RB** | interleukin 2 receptor subunit beta |
| 430. | **IL2RG** | interleukin 2 receptor subunit gamma |
| 431. | **IL3** | interleukin 3 |
| 432. | **IL31RA** | interleukin 31 receptor A |
| 433. | **IL32** | interleukin 32 |
| 434. | **IL33** | interleukin 33 |
| 435. | **IL34** | interleukin 34 |
| 436. | **IL36A** | interleukin 36, alpha |
| 437. | **IL36B** | interleukin 36, beta |
| 438. | **IL36G** | interleukin 36, gamma |
| 439. | **IL36RN** | interleukin 36 receptor antagonist |
| 440. | **IL37** | interleukin 37 |
| 441. | **IL3RA** | interleukin 3 receptor subunit alpha |
| 442. | **IL4** | interleukin 4 |
| 443. | **IL4R** | interleukin 4 receptor |
| 444. | **IL5** | interleukin 5 |
| 445. | **IL5RA** | interleukin 5 receptor subunit alpha |
| 446. | **IL6** | interleukin 6 |
| 447. | **IL6R** | interleukin 6 receptor |
| 448. | **IL6ST** | interleukin 6 signal transducer |
| 449. | **IL7** | interleukin 7 |
| 450. | **IL7R** | interleukin 7 receptor |
| 451. | **IL9** | interleukin 9 |
| 452. | **IL9R** | interleukin 9 receptor |
| 453. | **INHA** | inhibin alpha subunit |
| 454. | **INHBA** | inhibin beta A subunit |
| 455. | **INHBB** | inhibin beta B subunit |
| 456. | **INS** | insulin |
| 457. | **IRAK2** | interleukin 1 receptor associated kinase 2 |
| 458. | **IRF1** | interferon regulatory factor 1 |
| 459. | **IRF3** | interferon regulatory factor 3 |
| 460. | **IRF4** | interferon regulatory factor 4 |
| 461. | **IRF5** | interferon regulatory factor 5 |
| 462. | **IRF7** | interferon regulatory factor 7 |
| 463. | **IRGM** | immunity related GTPase M |
| 464. | **ISL1** | ISL LIM homeobox 1 |
| 465. | **ITCH** | itchy E3 ubiquitin protein ligase |
| 466. | **ITGA2** | integrin subunit alpha 2 |
| 467. | **ITGAL** | integrin subunit alpha L |
| 468. | **ITGB2** | integrin subunit beta 2 |
| 469. | **ITGB6** | integrin subunit beta 6 |
| 470. | **ITIH4** | inter-alpha-trypsin inhibitor heavy chain family member 4 |
| 471. | **JAK2** | Janus kinase 2 |
| 472. | **JAM3** | junctional adhesion molecule 3 |
| 473. | **JUN** | Jun proto-oncogene, AP-1 transcription factor subunit |
| 474. | **KDM6B** | lysine demethylase 6B |
| 475. | **KEAP1** | kelch like ECH associated protein 1 |
| 476. | **KIT** | KIT proto-oncogene receptor tyrosine kinase |
| 477. | **KITLG** | KIT ligand |
| 478. | **KLF4** | Kruppel like factor 4 |
| 479. | **KLKB1** | kallikrein B1 |
| 480. | **KLRG1** | killer cell lectin like receptor G1 |
| 481. | **KNG1** | kininogen 1 |
| 482. | **KRT1** | keratin 1 |
| 483. | **KRT16** | keratin 16 |
| 484. | **LAT** | linker for activation of T-cells |
| 485. | **LBP** | lipopolysaccharide binding protein |
| 486. | **LEFTY1** | left-right determination factor 1 |
| 487. | **LEFTY2** | left-right determination factor 2 |
| 488. | **LEP** | leptin |
| 489. | **LEPR** | leptin receptor |
| 490. | **LGALS9** | galectin 9 |
| 491. | **LIAS** | lipoic acid synthetase |
| 492. | **LIF** | leukemia inhibitory factor |
| 493. | **LIFR** | leukemia inhibitory factor receptor alpha |
| 494. | **LIMK1** | LIM domain kinase 1 |
| 495. | **LIPA** | lipase A, lysosomal acid type |
| 496. | **LTA** | lymphotoxin alpha |
| 497. | **LTA4H** | leukotriene A4 hydrolase |
| 498. | **LTB** | lymphotoxin beta |
| 499. | **LTB4R** | leukotriene B4 receptor |
| 500. | **LTB4R2** | leukotriene B4 receptor 2 |
| 501. | **LTBR** | lymphotoxin beta receptor |
| 502. | **LXN** | latexin |
| 503. | **LY75** | lymphocyte antigen 75 |
| 504. | **LY86** | lymphocyte antigen 86 |
| 505. | **LY96** | lymphocyte antigen 96 |
| 506. | **LYN** | LYN proto-oncogene, Src family tyrosine kinase |
| 507. | **MAFF** | MAF bZIP transcription factor F |
| 508. | **MAFG** | MAF bZIP transcription factor G |
| 509. | **MAFK** | MAF bZIP transcription factor K |
| 510. | **MAP2K1** | mitogen-activated protein kinase kinase 1 |
| 511. | **MAP2K3** | mitogen-activated protein kinase kinase 3 |
| 512. | **MAP2K4** | mitogen-activated protein kinase kinase 4 |
| 513. | **MAP2K6** | mitogen-activated protein kinase kinase 6 |
| 514. | **MAP3K1** | mitogen-activated protein kinase kinase kinase 1 |
| 515. | **MAP3K5** | mitogen-activated protein kinase kinase kinase 5 |
| 516. | **MAP3K7** | mitogen-activated protein kinase kinase kinase 7 |
| 517. | **MAP3K9** | mitogen-activated protein kinase kinase kinase 9 |
| 518. | **MAPK1** | mitogen-activated protein kinase 1 |
| 519. | **MAPK13** | mitogen-activated protein kinase 13 |
| 520. | **MAPK14** | mitogen-activated protein kinase 14 |
| 521. | **MAPK3** | mitogen-activated protein kinase 3 |
| 522. | **MAPK7** | mitogen-activated protein kinase 7 |
| 523. | **MAPK8** | mitogen-activated protein kinase 8 |
| 524. | **MAPKAPK2** | mitogen-activated protein kinase-activated protein kinase 2 |
| 525. | **MAPKAPK5** | mitogen-activated protein kinase-activated protein kinase 5 |
| 526. | **MAS1** | MAS1 proto-oncogene, G protein-coupled receptor |
| 527. | **MASP1** | mannan binding lectin serine peptidase 1 |
| 528. | **MASP2** | mannan binding lectin serine peptidase 2 |
| 529. | **MAX** | MYC associated factor X |
| 530. | **MBL2** | mannose binding lectin 2 |
| 531. | **MDK** | midkine (neurite growth-promoting factor 2) |
| 532. | **MECOM** | MDS1 and EVI1 complex locus |
| 533. | **MEF2A** | myocyte enhancer factor 2A |
| 534. | **MEF2B** | myocyte enhancer factor 2B |
| 535. | **MEF2C** | myocyte enhancer factor 2C |
| 536. | **MEF2D** | myocyte enhancer factor 2D |
| 537. | **MEFV** | MEFV, pyrin innate immunity regulator |
| 538. | **MEP1B** | meprin A subunit beta |
| 539. | **METRNL** | meteorin like, glial cell differentiation regulator |
| 540. | **MGLL** | monoglyceride lipase |
| 541. | **MIF** | macrophage migration inhibitory factor (glycosylation-inhibiting factor) |
| 542. | **MKNK1** | MAP kinase interacting serine/threonine kinase 1 |
| 543. | **MMP25** | matrix metallopeptidase 25 |
| 544. | **MMP26** | matrix metallopeptidase 26 |
| 545. | **MMP3** | matrix metallopeptidase 3 |
| 546. | **MMP9** | matrix metallopeptidase 9 |
| 547. | **MPL** | MPL proto-oncogene, thrombopoietin receptor |
| 548. | **MRC1** | mannose receptor C-type 1 |
| 549. | **MRGPRX1** | MAS related GPR family member X1 |
| 550. | **MS4A2** | membrane spanning 4-domains A2 |
| 551. | **MSTN** | myostatin |
| 552. | **MTA1** | metastasis associated 1 |
| 553. | **MUC4** | mucin 4, cell surface associated |
| 554. | **MVK** | mevalonate kinase |
| 555. | **MX1** | MX dynamin like GTPase 1 |
| 556. | **MX2** | MX dynamin like GTPase 2 |
| 557. | **MYC** | v-myc avian myelocytomatosis viral oncogene homolog |
| 558. | **MYD88** | myeloid differentiation primary response 88 |
| 559. | **MYL2** | myosin light chain 2 |
| 560. | **MYLK3** | myosin light chain kinase 3 |
| 561. | **NAIP** | NLR family apoptosis inhibitory protein |
| 562. | **NAMPT** | nicotinamide phosphoribosyltransferase |
| 563. | **NCF1** | neutrophil cytosolic factor 1 |
| 564. | **NCR3** | natural cytotoxicity triggering receptor 3 |
| 565. | **NDFIP1** | Nedd4 family interacting protein 1 |
| 566. | **NDST1** | N-deacetylase and N-sulfotransferase 1 |
| 567. | **NFAM1** | NFAT activating protein with ITAM motif 1 |
| 568. | **NFATC3** | nuclear factor of activated T-cells 3 |
| 569. | **NFATC4** | nuclear factor of activated T-cells 4 |
| 570. | **NFE2L1** | nuclear factor, erythroid 2 like 1 |
| 571. | **NFE2L2** | nuclear factor, erythroid 2 like 2 |
| 572. | **NFKB1** | nuclear factor kappa B subunit 1 |
| 573. | **NFKB2** | nuclear factor kappa B subunit 2 |
| 574. | **NFKBID** | NFKB inhibitor delta |
| 575. | **NFKBIZ** | NFKB inhibitor zeta |
| 576. | **NFRKB** | nuclear factor related to kappaB binding protein |
| 577. | **NFX1** | nuclear transcription factor, X-box binding 1 |
| 578. | **NGFR** | nerve growth factor receptor |
| 579. | **NLRC4** | NLR family CARD domain containing 4 |
| 580. | **NLRP1** | NLR family pyrin domain containing 1 |
| 581. | **NLRP12** | NLR family pyrin domain containing 12 |
| 582. | **NLRP2** | NLR family pyrin domain containing 2 |
| 583. | **NLRP3** | NLR family pyrin domain containing 3 |
| 584. | **NLRP4** | NLR family pyrin domain containing 4 |
| 585. | **NLRP6** | NLR family pyrin domain containing 6 |
| 586. | **NLRX1** | NLR family member X1 |
| 587. | **NMI** | N-myc and STAT interactor |
| 588. | **NOD1** | nucleotide binding oligomerization domain containing 1 |
| 589. | **NOD2** | nucleotide binding oligomerization domain containing 2 |
| 590. | **NODAL** | nodal growth differentiation factor |
| 591. | **NOS2** | nitric oxide synthase 2 |
| 592. | **NOTCH1** | notch 1 |
| 593. | **NOTCH2** | notch 2 |
| 594. | **NOV** | nephroblastoma overexpressed |
| 595. | **NOX1** | NADPH oxidase 1 |
| 596. | **NOX4** | NADPH oxidase 4 |
| 597. | **NOX5** | NADPH oxidase 5 |
| 598. | **NPFF** | neuropeptide FF-amide peptide precursor |
| 599. | **NPY5R** | neuropeptide Y receptor Y5 |
| 600. | **NR1D2** | nuclear receptor subfamily 1 group D member 2 |
| 601. | **NR1H3** | nuclear receptor subfamily 1 group H member 3 |
| 602. | **NR3C1** | nuclear receptor subfamily 3 group C member 1 |
| 603. | **NRROS** | negative regulator of reactive oxygen species |
| 604. | **NT5E** | 5'-nucleotidase ecto |
| 605. | **NTRK2** | neurotrophic receptor tyrosine kinase 2 |
| 606. | **NUPR1** | nuclear protein 1, transcriptional regulator |
| 607. | **OAS2** | 2'-5'-oligoadenylate synthetase 2 |
| 608. | **OASL** | 2'-5'-oligoadenylate synthetase like |
| 609. | **ODAM** | odontogenic, ameloblast asssociated |
| 610. | **OGG1** | 8-oxoguanine DNA glycosylase |
| 611. | **OLR1** | oxidized low density lipoprotein receptor 1 |
| 612. | **OPRM1** | opioid receptor mu 1 |
| 613. | **ORM1** | orosomucoid 1 |
| 614. | **ORM2** | orosomucoid 2 |
| 615. | **OSM** | oncostatin M |
| 616. | **OSMR** | oncostatin M receptor |
| 617. | **OTUD7A** | OTU deubiquitinase 7A |
| 618. | **OTULIN** | OTU deubiquitinase with linear linkage specificity |
| 619. | **OXER1** | oxoeicosanoid receptor 1 |
| 620. | **P2RX1** | purinergic receptor P2X 1 |
| 621. | **P2RX7** | purinergic receptor P2X 7 |
| 622. | **PARK7** | Parkinsonism associated deglycase |
| 623. | **PARP4** | poly(ADP-ribose) polymerase family member 4 |
| 624. | **PBK** | PDZ binding kinase |
| 625. | **PDE2A** | phosphodiesterase 2A |
| 626. | **PDGFA** | platelet derived growth factor subunit A |
| 627. | **PDGFB** | platelet derived growth factor subunit B |
| 628. | **PER1** | period circadian clock 1 |
| 629. | **PF4** | platelet factor 4 |
| 630. | **PF4V1** | platelet factor 4 variant 1 |
| 631. | **PGLYRP1** | peptidoglycan recognition protein 1 |
| 632. | **PGLYRP2** | peptidoglycan recognition protein 2 |
| 633. | **PHB** | prohibitin |
| 634. | **PIK3AP1** | phosphoinositide-3-kinase adaptor protein 1 |
| 635. | **PIK3C2G** | phosphatidylinositol-4-phosphate 3-kinase catalytic subunit type 2 gamma |
| 636. | **PIK3CA** | phosphatidylinositol-4,5-bisphosphate 3-kinase catalytic subunit alpha |
| 637. | **PIK3CB** | phosphatidylinositol-4,5-bisphosphate 3-kinase catalytic subunit beta |
| 638. | **PIK3CD** | phosphatidylinositol-4,5-bisphosphate 3-kinase catalytic subunit delta |
| 639. | **PIK3CG** | phosphatidylinositol-4,5-bisphosphate 3-kinase catalytic subunit gamma |
| 640. | **PLA2G2A** | phospholipase A2 group IIA |
| 641. | **PLA2G2D** | phospholipase A2 group IID |
| 642. | **PLA2G2E** | phospholipase A2 group IIE |
| 643. | **PLA2G4A** | phospholipase A2 group IVA |
| 644. | **PLA2G4C** | phospholipase A2 group IVC |
| 645. | **PLA2G7** | phospholipase A2 group VII |
| 646. | **PLAA** | phospholipase A2 activating protein |
| 647. | **PLCB1** | phospholipase C beta 1 |
| 648. | **PLP1** | proteolipid protein 1 |
| 649. | **PLSCR1** | phospholipid scramblase 1 |
| 650. | **PNMA1** | paraneoplastic Ma antigen 1 |
| 651. | **POLB** | DNA polymerase beta |
| 652. | **PPARA** | peroxisome proliferator activated receptor alpha |
| 653. | **PPARD** | peroxisome proliferator activated receptor delta |
| 654. | **PPARG** | peroxisome proliferator activated receptor gamma |
| 655. | **PPBP** | pro-platelet basic protein |
| 656. | **PPP1R12B** | protein phosphatase 1 regulatory subunit 12B |
| 657. | **PRCP** | prolylcarboxypeptidase |
| 658. | **PRDX5** | peroxiredoxin 5 |
| 659. | **PREX1** | phosphatidylinositol-3,4,5-trisphosphate dependent Rac exchange factor 1 |
| 660. | **PRG2** | proteoglycan 2, pro eosinophil major basic protein |
| 661. | **PRG3** | proteoglycan 3, pro eosinophil major basic protein 2 |
| 662. | **PRKCA** | protein kinase C alpha |
| 663. | **PRKCB** | protein kinase C beta |
| 664. | **PRKCD** | protein kinase C delta |
| 665. | **PRKCQ** | protein kinase C theta |
| 666. | **PRKCZ** | protein kinase C zeta |
| 667. | **PRKD1** | protein kinase D1 |
| 668. | **PRL** | prolactin |
| 669. | **PRLR** | prolactin receptor |
| 670. | **PROC** | protein C, inactivator of coagulation factors Va and VIIIa |
| 671. | **PROCR** | protein C receptor |
| 672. | **PROK2** | prokineticin 2 |
| 673. | **PROS1** | protein S (alpha) |
| 674. | **PSMA6** | proteasome subunit alpha 6 |
| 675. | **PSMB4** | proteasome subunit beta 4 |
| 676. | **PSTPIP1** | proline-serine-threonine phosphatase interacting protein 1 |
| 677. | **PTAFR** | platelet activating factor receptor |
| 678. | **PTGDR** | prostaglandin D2 receptor |
| 679. | **PTGDR2** | prostaglandin D2 receptor 2 |
| 680. | **PTGER1** | prostaglandin E receptor 1 |
| 681. | **PTGER2** | prostaglandin E receptor 2 |
| 682. | **PTGER3** | prostaglandin E receptor 3 |
| 683. | **PTGER4** | prostaglandin E receptor 4 |
| 684. | **PTGES** | prostaglandin E synthase |
| 685. | **PTGFR** | prostaglandin F receptor |
| 686. | **PTGIR** | prostaglandin I2 (prostacyclin) receptor (IP) |
| 687. | **PTGIS** | prostaglandin I2 synthase |
| 688. | **PTGS1** | prostaglandin-endoperoxide synthase 1 |
| 689. | **PTGS2** | prostaglandin-endoperoxide synthase 2 |
| 690. | **PTK2** | protein tyrosine kinase 2 |
| 691. | **PTN** | pleiotrophin |
| 692. | **PTPN2** | protein tyrosine phosphatase, non-receptor type 2 |
| 693. | **PTPRA** | protein tyrosine phosphatase, receptor type A |
| 694. | **PTX3** | pentraxin 3 |
| 695. | **PXK** | PX domain containing serine/threonine kinase like |
| 696. | **PXMP2** | peroxisomal membrane protein 2 |
| 697. | **PYCARD** | PYD and CARD domain containing |
| 698. | **PYDC2** | pyrin domain containing 2 |
| 699. | **RABGEF1** | RAB guanine nucleotide exchange factor 1 |
| 700. | **RAC1** | ras-related C3 botulinum toxin substrate 1 (rho family, small GTP binding protein Rac1) |
| 701. | **RAF1** | Raf-1 proto-oncogene, serine/threonine kinase |
| 702. | **RAPGEF2** | Rap guanine nucleotide exchange factor 2 |
| 703. | **RARRES2** | retinoic acid receptor responder 2 |
| 704. | **RASGRP1** | RAS guanyl releasing protein 1 |
| 705. | **RBPJ** | recombination signal binding protein for immunoglobulin kappa J region |
| 706. | **REG3A** | regenerating family member 3 alpha |
| 707. | **REG3G** | regenerating family member 3 gamma |
| 708. | **REL** | REL proto-oncogene, NF-kB subunit |
| 709. | **RELA** | RELA proto-oncogene, NF-kB subunit |
| 710. | **RELB** | RELB proto-oncogene, NF-kB subunit |
| 711. | **RELT** | RELT tumor necrosis factor receptor |
| 712. | **RHBDD3** | rhomboid domain containing 3 |
| 713. | **RHOA** | ras homolog family member A |
| 714. | **RICTOR** | RPTOR independent companion of MTOR complex 2 |
| 715. | **RIPK1** | receptor interacting serine/threonine kinase 1 |
| 716. | **RIPK2** | receptor interacting serine/threonine kinase 2 |
| 717. | **ROCK2** | Rho associated coiled-coil containing protein kinase 2 |
| 718. | **RORA** | RAR related orphan receptor A |
| 719. | **RPS19** | ribosomal protein S19 |
| 720. | **RPS6KA4** | ribosomal protein S6 kinase A4 |
| 721. | **RPS6KA5** | ribosomal protein S6 kinase A5 |
| 722. | **RXRA** | retinoid X receptor alpha |
| 723. | **S100A12** | S100 calcium binding protein A12 |
| 724. | **S100A8** | S100 calcium binding protein A8 |
| 725. | **S100A9** | S100 calcium binding protein A9 |
| 726. | **S100B** | S100 calcium binding protein B |
| 727. | **S1PR3** | sphingosine-1-phosphate receptor 3 |
| 728. | **SAA1** | serum amyloid A1 |
| 729. | **SAA2** | serum amyloid A2 |
| 730. | **SAA4** | serum amyloid A4, constitutive |
| 731. | **SBNO2** | strawberry notch homolog 2 |
| 732. | **SCG2** | secretogranin II |
| 733. | **SCGB1A1** | secretoglobin family 1A member 1 |
| 734. | **SCN9A** | sodium voltage-gated channel alpha subunit 9 |
| 735. | **SCUBE1** | signal peptide, CUB domain and EGF like domain containing 1 |
| 736. | **SDC1** | syndecan 1 |
| 737. | **SDCBP** | syndecan binding protein |
| 738. | **SECTM1** | secreted and transmembrane 1 |
| 739. | **SEH1L** | SEH1 like nucleoporin |
| 740. | **SELE** | selectin E |
| 741. | **SELENOS** | selenoprotein S |
| 742. | **SELP** | selectin P |
| 743. | **SEMA7A** | semaphorin 7A (John Milton Hagen blood group) |
| 744. | **SERPINA1** | serpin family A member 1 |
| 745. | **SERPINA3** | serpin family A member 3 |
| 746. | **SERPINC1** | serpin family C member 1 |
| 747. | **SERPINE1** | serpin family E member 1 |
| 748. | **SERPINF1** | serpin family F member 1 |
| 749. | **SERPINF2** | serpin family F member 2 |
| 750. | **SERPING1** | serpin family G member 1 |
| 751. | **SETD6** | SET domain containing 6 |
| 752. | **SFTPD** | surfactant protein D |
| 753. | **SHARPIN** | SHANK associated RH domain interactor |
| 754. | **SHC1** | SHC adaptor protein 1 |
| 755. | **SHPK** | sedoheptulokinase |
| 756. | **SIGIRR** | single Ig and TIR domain containing |
| 757. | **SIGLEC1** | sialic acid binding Ig like lectin 1 |
| 758. | **SLC11A1** | solute carrier family 11 member 1 |
| 759. | **SLC7A2** | solute carrier family 7 member 2 |
| 760. | **SLCO1A2** | solute carrier organic anion transporter family member 1A2 |
| 761. | **SLIT2** | slit guidance ligand 2 |
| 762. | **SLURP1** | secreted LY6/PLAUR domain containing 1 |
| 763. | **SMAD1** | SMAD family member 1 |
| 764. | **SMAD3** | SMAD family member 3 |
| 765. | **SMAD7** | SMAD family member 7 |
| 766. | **SNAP23** | synaptosome associated protein 23 |
| 767. | **SNX4** | sorting nexin 4 |
| 768. | **SOCS2** | suppressor of cytokine signaling 2 |
| 769. | **SOCS3** | suppressor of cytokine signaling 3 |
| 770. | **SP100** | SP100 nuclear antigen |
| 771. | **SPACA3** | sperm acrosome associated 3 |
| 772. | **SPHK1** | sphingosine kinase 1 |
| 773. | **SPN** | sialophorin |
| 774. | **SPP1** | secreted phosphoprotein 1 |
| 775. | **SPRED1** | sprouty related EVH1 domain containing 1 |
| 776. | **SRGAP1** | SLIT-ROBO Rho GTPase activating protein 1 |
| 777. | **STAB1** | stabilin 1 |
| 778. | **STAT1** | signal transducer and activator of transcription 1 |
| 779. | **STAT2** | signal transducer and activator of transcription 2 |
| 780. | **STAT3** | signal transducer and activator of transcription 3 |
| 781. | **STAT5A** | signal transducer and activator of transcription 5A |
| 782. | **STAT5B** | signal transducer and activator of transcription 5B |
| 783. | **STK39** | serine/threonine kinase 39 |
| 784. | **SUSD4** | sushi domain containing 4 |
| 785. | **SYK** | spleen associated tyrosine kinase |
| 786. | **TAC1** | tachykinin precursor 1 |
| 787. | **TAC4** | tachykinin 4 (hemokinin) |
| 788. | **TACR1** | tachykinin receptor 1 |
| 789. | **TBC1D23** | TBC1 domain family member 23 |
| 790. | **TBK1** | TANK binding kinase 1 |
| 791. | **TBXA2R** | thromboxane A2 receptor |
| 792. | **TCF4** | transcription factor 4 |
| 793. | **TEK** | TEK receptor tyrosine kinase |
| 794. | **TFF2** | trefoil factor 2 |
| 795. | **TFRC** | transferrin receptor |
| 796. | **TGFB1** | transforming growth factor beta 1 |
| 797. | **TGFB2** | transforming growth factor beta 2 |
| 798. | **TGFB3** | transforming growth factor beta 3 |
| 799. | **TGFBR1** | transforming growth factor beta receptor 1 |
| 800. | **THBS1** | thrombospondin 1 |
| 801. | **THPO** | thrombopoietin |
| 802. | **TICAM1** | toll like receptor adaptor molecule 1 |
| 803. | **TIRAP** | TIR domain containing adaptor protein |
| 804. | **TLR1** | toll like receptor 1 |
| 805. | **TLR10** | toll like receptor 10 |
| 806. | **TLR2** | toll like receptor 2 |
| 807. | **TLR3** | toll like receptor 3 |
| 808. | **TLR4** | toll like receptor 4 |
| 809. | **TLR5** | toll like receptor 5 |
| 810. | **TLR6** | toll like receptor 6 |
| 811. | **TLR7** | toll like receptor 7 |
| 812. | **TLR8** | toll like receptor 8 |
| 813. | **TLR9** | toll like receptor 9 |
| 814. | **TNF** | tumor necrosis factor |
| 815. | **TNFAIP3** | TNF alpha induced protein 3 |
| 816. | **TNFAIP6** | TNF alpha induced protein 6 |
| 817. | **TNFAIP8L2** | TNF alpha induced protein 8 like 2 |
| 818. | **TNFRSF10A** | TNF receptor superfamily member 10a |
| 819. | **TNFRSF10B** | TNF receptor superfamily member 10b |
| 820. | **TNFRSF10C** | TNF receptor superfamily member 10c |
| 821. | **TNFRSF10D** | TNF receptor superfamily member 10d |
| 822. | **TNFRSF11A** | TNF receptor superfamily member 11a |
| 823. | **TNFRSF11B** | TNF receptor superfamily member 11b |
| 824. | **TNFRSF14** | TNF receptor superfamily member 14 |
| 825. | **TNFRSF18** | TNF receptor superfamily member 18 |
| 826. | **TNFRSF1A** | TNF receptor superfamily member 1A |
| 827. | **TNFRSF1B** | TNF receptor superfamily member 1B |
| 828. | **TNFRSF21** | TNF receptor superfamily member 21 |
| 829. | **TNFRSF25** | TNF receptor superfamily member 25 |
| 830. | **TNFRSF4** | TNF receptor superfamily member 4 |
| 831. | **TNFRSF6B** | TNF receptor superfamily member 6b |
| 832. | **TNFRSF8** | TNF receptor superfamily member 8 |
| 833. | **TNFRSF9** | TNF receptor superfamily member 9 |
| 834. | **TNFSF10** | tumor necrosis factor superfamily member 10 |
| 835. | **TNFSF11** | tumor necrosis factor superfamily member 11 |
| 836. | **TNFSF13** | tumor necrosis factor superfamily member 13 |
| 837. | **TNFSF13B** | tumor necrosis factor superfamily member 13b |
| 838. | **TNFSF14** | tumor necrosis factor superfamily member 14 |
| 839. | **TNFSF15** | tumor necrosis factor superfamily member 15 |
| 840. | **TNFSF18** | tumor necrosis factor superfamily member 18 |
| 841. | **TNFSF4** | tumor necrosis factor superfamily member 4 |
| 842. | **TNFSF8** | tumor necrosis factor superfamily member 8 |
| 843. | **TNFSF9** | tumor necrosis factor superfamily member 9 |
| 844. | **TNIP1** | TNFAIP3 interacting protein 1 |
| 845. | **TNIP2** | TNFAIP3 interacting protein 2 |
| 846. | **TNIP3** | TNFAIP3 interacting protein 3 |
| 847. | **TOLLIP** | toll interacting protein |
| 848. | **TP73** | tumor protein p73 |
| 849. | **TPST1** | tyrosylprotein sulfotransferase 1 |
| 850. | **TRADD** | TNFRSF1A associated via death domain |
| 851. | **TRAF2** | TNF receptor associated factor 2 |
| 852. | **TRAP1** | TNF receptor associated protein 1 |
| 853. | **TREM2** | triggering receptor expressed on myeloid cells 2 |
| 854. | **TRIL** | TLR4 interactor with leucine rich repeats |
| 855. | **TRPV1** | transient receptor potential cation channel subfamily V member 1 |
| 856. | **TSLP** | thymic stromal lymphopoietin |
| 857. | **TSPAN2** | tetraspanin 2 |
| 858. | **TTN** | titin |
| 859. | **TUSC2** | tumor suppressor candidate 2 |
| 860. | **TWIST2** | twist family bHLH transcription factor 2 |
| 861. | **TYMP** | thymidine phosphorylase |
| 862. | **TYRO3** | TYRO3 protein tyrosine kinase |
| 863. | **TYROBP** | TYRO protein tyrosine kinase binding protein |
| 864. | **UCN** | urocortin |
| 865. | **UNC13D** | unc-13 homolog D |
| 866. | **VAMP7** | vesicle associated membrane protein 7 |
| 867. | **VAMP8** | vesicle associated membrane protein 8 |
| 868. | **VCAM1** | vascular cell adhesion molecule 1 |
| 869. | **VEGFA** | vascular endothelial growth factor A |
| 870. | **VEGFB** | vascular endothelial growth factor B |
| 871. | **VEGFD** | vascular endothelial growth factor D |
| 872. | **VNN1** | vanin 1 |
| 873. | **VPS45** | vacuolar protein sorting 45 homolog |
| 874. | **VTN** | vitronectin |
| 875. | **WFDC1** | WAP four-disulfide core domain 1 |
| 876. | **WNT5A** | Wnt family member 5A |
| 877. | **XCL1** | X-C motif chemokine ligand 1 |
| 878. | **XCR1** | X-C motif chemokine receptor 1 |
| 879. | **XIAP** | X-linked inhibitor of apoptosis |
| 880. | **YARS** | tyrosyl-tRNA synthetase |
| 881. | **ZC3H12A** | zinc finger CCCH-type containing 12A |
| 882. | **ZNF580** | zinc finger protein 580 |
| 883. | **ZP3** | zona pellucida glycoprotein 3 |
| 884. | **ZYX** | zyxin |
